# The pnictogen bond: a quantitative molecular orbital picture†

**DOI:** 10.1039/d1cp01571k

**Published:** 2021-06-04

**Authors:** Lucas de Azevedo Santos, Trevor A. Hamlin, Teodorico C. Ramalho, F. Matthias Bickelhaupt

**Affiliations:** Department of Theoretical Chemistry, Amsterdam Institute for Molecular and Life Sciences (AIMMS), Amsterdam Center for Multiscale Modeling (ACMM), Vrije Universiteit Amsterdam De Boelelaan 1083 1081 HV Amsterdam The Netherlands f.m.bickelhaupt@vu.nl; Department of Chemistry, Institute of Natural Sciences, Universidade Federal de Lavras 37200-900 Lavras MG Brazil; Center for Basic and Applied Research, University Hradec Kralove Hradec Kralove Czech Republic; Institute for Molecules and Materials, Radboud University Nijmegen Heyendaalseweg 135 6525 AJ Nijmegen The Netherlands

## Abstract

We have analyzed the structure and stability of archetypal pnictogen-bonded model complexes D_3_Pn⋯A^−^ (Pn = N, P, As, Sb; D, A = F, Cl, Br) using state-of-the-art relativistic density functional calculations at the ZORA-M06/QZ4P level. We have accomplished two tasks: (i) to compute accurate trends in pnictogen-bond strength based on a set of consistent data; and (ii) to rationalize these trends in terms of detailed analyses of the bonding mechanism based on quantitative Kohn–Sham molecular orbital (KS-MO) theory in combination with a canonical energy decomposition analysis (EDA) and Voronoi deformation density (VDD) analyses of the charge distribution. We have found that pnictogen bonds have a significant covalent character stemming from strong HOMO–LUMO interactions between the lone pair of A^−^ and σ* of D_3_Pn. As such, the underlying mechanism of the pnictogen bond is similar to that of hydrogen, halogen, and chalcogen bonds.

## Abstract 2

Our quantitative molecular orbital analyses show that pnictogen bonds are not solely electrostatic phenomena, but also have a strongly stabilizing covalent component, just like chalcogen-, halogen-, and hydrogen bonds.

## Graphical abstract

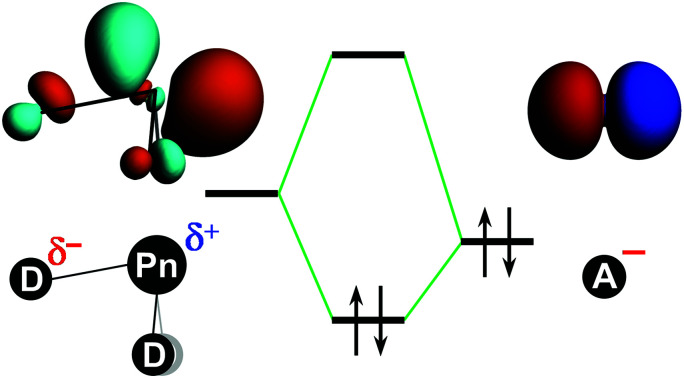

## Introduction

The term pnictogen for the elements of the nitrogen group (group 15) was first proposed by van Arkel in the early 1950s.^1^ Its etymology derives from the Ancient Greek root πνιγ (“choke”) and is a reference to the Dutch and German names for nitrogen, stikstof and Stickstoff, respectively, which literally mean “suffocation substance”. The trivalent pnictogen atom of a Lewis-acidic pnictogen-bond donor D_3_Pn (Pn = group 15 atom) can engage in an intermolecular interaction, coined a pnictogen bond, with a Lewis-basic pnictogen-bond acceptor A^−^.^2^ One of the first indications of Pn bonding appeared from stacked distibines and dibismuthines in crystal structures^3*a*^ and from intramolecular N⋯P contacts in hypervalent phosphorus compounds.^3*b*^ Later, a weak P⋯P interaction was identified *via* through-space coupling by NMR of phosphanyl-*ortho*-carbaboranes.^3*c*–*e*^ Since then, Pn bonding has flourished and emerged as a tool for coordination chemistry^4^ and catalysis.^5^ The nature of pnictogen bonds (similar to that of chalcogen and halogen bonds) is in general considered predominantly electrostatic,^6^ although the bonding mechanism of weak interactions is still under debate.^2*b*,7,8^

**Scheme 1:**
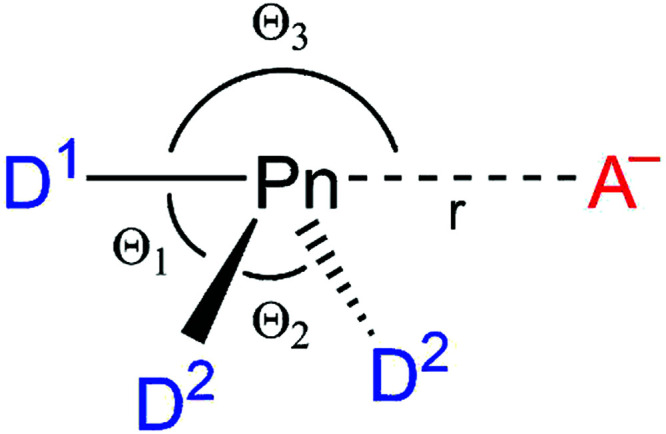
Pnictogen-bonded D_3_Pn⋯A^−^ model complexes (Pn = N, P, As, Sb; D, A = F, Cl, Br).

In this study, we have computationally analyzed a range of pnictogen-bonded D_3_Pn⋯A^−^ complexes (Pn = N, P, As, Sb; D, A = F, Cl, Br; see Scheme 1), using relativistic density functional theory (DFT) at the ZORA-M06/QZ4P level. One purpose of our work is to provide a set of consistent structural and energy data from which reliable trends can be inferred for a wide range of model systems. From these data, we have constructed a unified framework to rationalize the nature of pnictogen bonds, chalcogen bonds, halogen bonds, and hydrogen bonds, by studying the associated electronic structure and bonding mechanism.^8^

To this end, the pnictogen atom (Pn), the substituent (D), or the pnictogen bond accepting Lewis base (A^−^) are systematically varied to assess how the geometries and energies of our model complexes D_3_Pn⋯A^−^ are affected. Activation strain analyses^9^ are performed on the formation of the pnictogen-bond complexes to understand the origin of the computed trends. As part of these analyses, the underlying bonding mechanism is elucidated in the context of Kohn–Sham molecular orbital (MO) theory in combination with a matching energy decomposition analysis (EDA) as implemented in the Amsterdam Density Functional (ADF) program.^10,11^ Our analyses along the entire reaction profile for each of the pnictogen-bond complexation reactions demonstrate that pnictogen bonds are not at all purely electrostatic phenomena. Instead, they are, to a substantial extent, covalent in nature, very similar to chalcogen bonds, halogen bonds, and hydrogen bonds.

## Theoretical methods

### Computational details

All calculations were carried out using the Amsterdam Density Functional (ADF) 2017.103 program.^11^ The equilibrium geometries and energies of pnictogen-bonded complexes were computed at the DFT level using the meta-hybrid functional M06.^12^ A large uncontracted relativistically optimized QZ4P Slater type orbital (STO) basis set containing diffuse functions was used. The QZ4P all-electron basis set,^13^ no frozen-core approximation, is of quadruple-*ζ* quality for all atoms and has been augmented with the following sets of polarization and diffuse functions: two 3d and two 4f on nitrogen and fluorine, three 3d and two 4f on phosphorus and chlorine, two 4d and three 4f on arsenic and bromine, and one 5d and three 4f on antimony and iodine. The molecular density was fitted by the systematically improvable Zlm fitting scheme. Scalar relativistic effects were accounted for using the zeroth-order regular approximation (ZORA) Hamiltonian.^14^

### Analysis of the bonding mechanism

Insight into the bonding mechanism is obtained through activation strain analyses of the various pnictogen bond formation reactions. These complexation reactions are computationally modeled by decreasing the distance between A^−^ and the Pn atom of the D_3_Pn fragment, allowing the system to geometrically relax at each point. The D_3_Pn⋯A^−^ distance is increased, starting from the equilibrium geometry in the pnictogen-bonded complex (*r*_Pn⋯A_), to a value of 5.300 Å. Thus, each analysis starts from an optimized D_3_Pn⋯A^−^ complex, which is then transformed into the D_3_Pn molecule and a halide at a relatively large distance.

These complexation reactions are analyzed using the activation strain model. The activation strain model of chemical reactivity^9^ is a fragment-based approach to understand the energy profile of a chemical process in terms of the original reactants. Thus, the potential energy surface Δ*E*(*ζ*) is decomposed along the reaction coordinate *ζ* (or just at one point along *ζ*) into the strain energy Δ*E*_strain_(*ζ*), which is associated with the geometrical deformation of the individual reactants as the process takes place, plus the actual interaction energy Δ*E*_int_(*ζ*) between the deformed reactants [eqn (1)].

1 Δ*E*(*ζ*) = Δ*E*_strain_(*ζ*) + Δ*E*_int_(*ζ*)

In the equilibrium geometry, that is, for *ζ* = *ζ*_eq_, this yields an expression for the bond energy Δ*E*(*ζ*_eq_) = Δ*E*_strain_ + Δ*E*_int_. The PyFrag program was used to facilitate the analyses along the reaction coordinate *ζ* of the bond formation processes.^15^ The interaction energy Δ*E*_int_(*ζ*) between the deformed reactants is further analyzed in the conceptual framework provided by the quantitative Kohn–Sham MO model.^10^ To this end, it is decomposed into three physically meaningful terms [eqn (2)] using a quantitative energy decomposition analysis (EDA) as implemented in ADF.^10,11^

2 Δ*E*_int_(*ζ*) = Δ*V*_elstat_(*ζ*) + Δ*E*_Pauli_(*ζ*) +Δ*E*_oi_(*ζ*)

The usually attractive term Δ*V*_elstat_ corresponds to the classical Coulomb interaction between the unperturbed charge distributions of the deformed reactants and has four components [eqn (3)]: (i) the electrostatic repulsion between the electron densities of fragments 1 and 2, Δ*V*_elstat,ρ_1_ρ_2__; (ii) the electrostatic attraction between the nucleus of fragment 1 and the electron density of fragment 2, Δ*V*_elstat,n_1_ρ_2__; (iii) the electrostatic attraction between the electron density of fragment 1 and the nucleus of fragment 2, Δ*V*_elstat,ρ_1_n_2__; and (iv) the electrostatic repulsion between the nuclei of fragments 1 and 2, Δ*V*_elstat,n_1_n_2__.

3 Δ*V*_elstat_(*ζ*) = Δ*V*_elstat,ρ_1_ρ_2__(*ζ*) + Δ*V*_elstat,n_1_ρ_2__(*ζ*) + Δ*V*_elstat,ρ_1_n_2__(*ζ*) + Δ*V*_elstat,n_1_n_2__(*ζ*)

The Pauli repulsion energy (Δ*E*_Pauli_) comprises the destabilizing interactions between occupied orbitals of one reactant and those of another reactant and is responsible for steric repulsion. The orbital-interaction energy (Δ*E*_oi_) accounts for charge transfer, that is, the interaction between occupied orbitals of one fragment and unoccupied orbitals of the other fragment, including the interactions of the highest occupied and lowest unoccupied MOs (HOMO–LUMO), and polarization, that is, empty–occupied orbital mixing on one fragment, due to the presence of another fragment.

The electron density distribution is analyzed using the Voronoi deformation density (VDD) method for computing atomic charges.^16^ The VDD atomic charge on atom X in a molecule (*Q*^VDD^_X_) is computed as the (numerical) integral of the deformation density in the volume of the Voronoi cell of atom X [eqn (4)]. The Voronoi cell of atom X is defined as the compartment of space bounded by the bond midplanes on and perpendicular to all bond axes between nucleus X and its neighboring nuclei.4
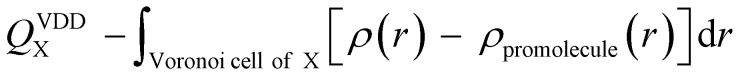


Here, the deformation density is the difference between *ρ*(*r*), *i.e.*, the electron density of the overall molecule or complex, and *ρ*_promolecule_(*r*) = *∑*_Y_*ρ*_Y_(r), *i.e.*, the superposition of spherical average-of-configuration atomic densities *ρ*_Y_(*r*) of each atom *Y* in the fictitious promolecule without chemical interactions, in which all atoms are considered neutral. The interpretation of the VDD charge *Q*^VDD^_Pn_ is rather straightforward and transparent: instead of measuring the amount of charge associated with a particular atom Pn, *Q*^VDD^_Pn_ directly monitors how much charge flows out of (*Q*^VDD^_Pn_ > 0) or into (*Q*^VDD^_Pn_ < 0) the Voronoi cell of atom Pn due to chemical interactions.

The VDD scheme can also be used to directly compute how much charge flows into or out of an atomic Voronoi cell X in an overall complex (*e.g.*, [D_3_Pn⋯A]^−^) relative to two (poly)atomic molecular fragments (*e.g.*, D_3_Pn and A^−^), instead of spherical atoms, as shown in eqn (5).5
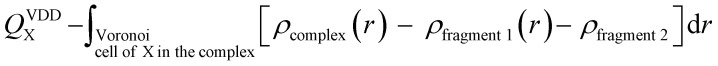


Δ*Q*^VDD^_X_ is a measure of how the atomic charge of atom X changes due to the bonding between the fragments. In this work, eqn (5) is used to compute the flow of electrons from the halide A^−^ to the pnictogen-bond donating molecule D_3_Pn (see 
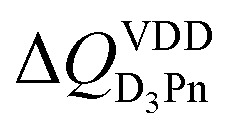
 in Tables 1 and 3).

**Table 1:** Activation strain analyses (in kcal mol^−1^) of a representative set of D_3_Pn⋯A^−^ at the equilibrium geometries (in Å, deg.)^a^

D_3_Pn⋯A^−^	Δ*E*	Δ*E*_strain_	Δ*E*_int_	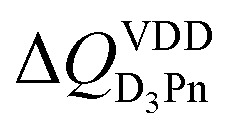	*r*_Pn⋯A_	Δ*r*_D^1^–Pn_	Δ*r*_D^2^–Pn_	*Θ*_3_	Δ*Θ*_1_
F_3_N⋯F^−^	−11.8	32.5	−44.3	−0.30	1.859	0.503	−0.017	170.3	−8.9
F_3_N⋯Cl^−^	−3.5	0.8	−4.3	−0.01	3.239	0.048	−0.009	168.3	−1.5
F_3_N⋯Br^−^	−2.9	0.5	−3.5	0.00	3.484	0.040	−0.008	166.8	−1.2
Cl_3_N⋯F^−^	−30.4	55.7	−86.1	−0.65	1.416	1.490	0.001	170.7	−25.7
Cl_3_N⋯Cl^−^	−5.6	22.9	−28.5	−0.36	2.328	0.575	0.004	146.6	−7.8
Cl_3_N⋯Br^−^	−6.2	3.5	−9.7	−0.18	2.920	0.149	0.024	145.6	−2.8
Br_3_N⋯F^−^	−30.2	53.2	−83.3	−0.67	1.411	1.417	0.022	165.7	−24.0
Br_3_N⋯Cl^−^	−8.0	2.0	−10.0	−0.18	2.813	0.111	0.026	149.4	−2.8
Br_3_N⋯Br^−^	−7.0	17.2	−24.2	−0.44	2.322	0.416	0.066	127.2	−3.1
F_3_P⋯F^−^	−48.9	17.4	−66.4	−0.35	1.753	0.189	0.044	189.4	−10.6
F_3_P⋯Cl^−^	−16.0	4.4	−20.4	−0.12	2.700	0.083	0.016	183.4	−5.6
F_3_P⋯Br^−^	−12.9	3.0	−15.9	−0.09	2.982	0.067	0.012	181.6	−4.6
Cl_3_P⋯F^−^	−67.4	31.8	−99.3	−0.52	1.649	0.572	0.048	181.4	−11.8
Cl_3_P⋯Cl^−^	−25.5	14.4	−39.8	−0.31	2.370	0.315	0.034	174.6	−8.7
Cl_3_P⋯Br^−^	−20.5	10.7	−31.3	−0.26	2.617	0.263	0.030	172.9	−7.6
Br_3_P⋯F^−^	−71.0	30.2	−101.2	−0.56	1.637	0.647	0.045	175.4	−11.3
Br_3_P⋯Cl^−^	−28.5	15.4	−43.9	−0.37	2.312	0.382	0.038	172.0	−8.9
Br_3_P⋯Br^−^	−23.4	11.9	−35.3	−0.32	2.550	0.323	0.034	170.6	−7.9
F_3_Sb⋯F^−^	−72.0	8.8	−80.8	−0.31	2.037	0.144	0.037	193.9	−8.9
F_3_Sb⋯Cl^−^	−38.9	5.8	−44.7	−0.21	2.643	0.112	0.030	188.3	−7.3
F_3_Sb⋯Br^−^	−33.6	5.0	−38.7	−0.19	2.840	0.103	0.028	186.7	−6.8
Cl_3_Sb⋯F^−^	−77.1	12.3	−89.4	−0.38	2.017	0.301	0.051	183.7	−7.6
Cl_3_Sb⋯Cl^−^	−42.7	8.9	−51.7	−0.28	2.592	0.244	0.045	178.4	−6.5
Cl_3_Sb⋯Br^−^	−37.3	8.0	−45.3	−0.27	2.780	0.229	0.043	177.0	−6.1
Br_3_Sb⋯F^−^	−77.7	11.5	−89.2	−0.41	2.014	0.332	0.049	181.2	−7.2
Br_3_Sb⋯Cl^−^	−43.5	8.5	−52.0	−0.31	2.580	0.272	0.046	175.6	−6.1
Br_3_Sb⋯Br^−^	−38.1	7.7	−45.8	−0.30	2.766	0.256	0.045	174.2	−5.8

a Computed at the ZORA-M06/QZ4P level. For a full set of data, see Tables S1 and S2 in the ESI.

## Results and discussion

### Pnictogen bond strength and structure

Table 1 summarizes the results of our ZORA-M06/QZ4P calculations for a representative selection of nitrogen-, phosphorus-, and antimony-bonded model complexes D_3_Pn⋯A^−^, covering D, A = F, Cl, and Br (for the complete dataset see Tables S1 and S2, ESI†). These model reactions go with a single-well potential energy surface (PES), that is, there is no energy barrier separating the reactants from their resulting product. In the cases where D ≠ A, *C*_S_ symmetric complexes with D^1^–Pn bond lengths different from the Pn⋯A^−^ bond and with bond angles *Θ*_1_ ≠ *Θ*_2_ are formed. For the cases where D = A, *C*_2v_ symmetric complexes with equal bond distances *r*_D^1^–Pn_ = *r*_Pn⋯A_ are formed (see Table 1).

The pnictogen bonds D_3_Pn⋯A^−^ become stronger and longer upon descending group 15 in the periodic table, going from N to Sb. The pnictogen bonds become weaker and longer as the accepting halide (A^−^) goes down group 17, from F^−^ to Br^−^. The elongation of the bonds descending the periodic table originates from the increase in the effective size of the atoms involved. In the case of the antimony-bonded complexes Br_3_Sb⋯A^−^, for example, Δ*E* weakens from a value of −78 kcal mol^−1^ for A^−^ = F^−^ to −38 kcal mol^−1^ for A^−^ = Br^−^ (see Table 1). The associated Sb⋯A^−^ bond elongates from around 2.0 Å for A^−^ = F^−^ to around 2.8 Å for A^−^ = Br^−^. From A^−^ = F^−^ to Br^−^, the nitrogen bond in Br_3_N⋯A^−^ weakens from a value of −30 kcal mol^−1^ to −7 kcal mol^−1^. The associated N⋯A^−^ bond elongates from a value of around 1.4 Å for A^−^ = F^−^ to around 2.3 Å for A^−^ = Br^−^. The reason behind the trends in stability will be discussed later.

The strengths of the heavier pnictogen bonds D_3_Pn⋯A^−^ are minimally affected upon variation of the substituent D. For example, along the series from F_3_Sb⋯F^−^ to Br_3_Sb⋯F^−^, the bond strength varies only from −72.0 to −77.7 kcal mol^−1^, the antimony bond distance *r*_Pn⋯A_ decreases slightly from 2.037 to 2.014 Å, and the stretch Δ*r*_D^1^–Pn_ upon bond formation increases from 0.144 to 0.332 Å. The nitrogen bonds D_3_N⋯A^−^ behave differently and become significantly stronger and shorter as D is varied from F to Br (see Table 1). For example, along the series from F_3_N⋯F^−^ to Br_3_N⋯F^−^, the nitrogen bond strengthens from a Δ*E* value of −11.8 to −30.2 kcal mol^−1^, the nitrogen bond distance *r*_Pn⋯A_ decreases in value from 1.859 to 1.411 Å, and the stretch Δ*r*_D^1^–Pn_ significantly increases from 0.503 to 1.417 Å. A comprehensive analysis of the origin of these trends is provided in the following.

### Bond analyses with the variation of Pn

**Fig. 1:**
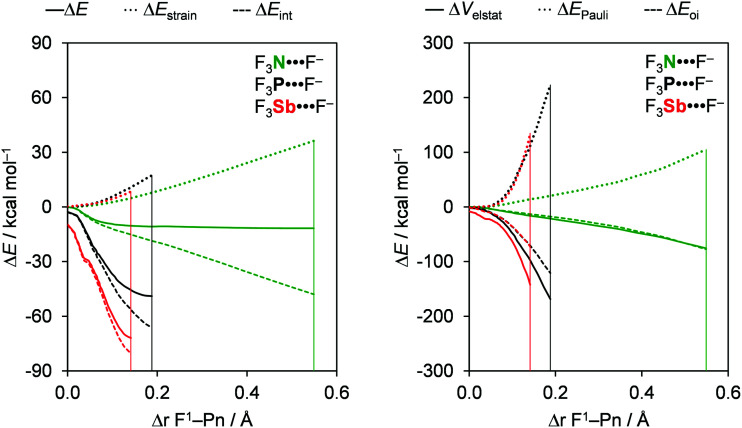
Activation strain (left panel) and energy decomposition (right panel) analyses of a representative set of F_3_Pn⋯F^−^ pnictogen-bonded complexes (green, Pn = N; black, Pn = P; red, Pn = Sb). The vertical lines indicate the position of the stationary points. For a full set of data, see Fig. S2 in the ESI.†

The pnictogen bond D_3_Pn⋯A^−^ strength Δ*E* increases as Pn varies along N, P, As, and Sb when the donating atom (D) and the accepting halide (A^−^) remain unchanged and the trend in Δ*E* is mainly set by the interaction energy Δ*E*_int_. For example, from F_3_N⋯F^−^ to F_3_Sb⋯F^−^, Δ*E* is strengthened from a value of −11.8 to −72.0 kcal mol^−1^ and Δ*E*_int_ is strengthened from a value of −44.3 to −80.8 kcal mol^−1^ (see Table 1). The trend in Δ*E* is reinforced by the strain energy (Δ*E*_strain_), which becomes less destabilizing from 32.5 to 8.8 kcal mol^−1^ along the same series. We extend our analysis to the entire reaction coordinate *ζ*, projected onto the stretch in the D^1^–Pn bond, Δ*r*_D^1^–Pn_, that occurs as the pnictogen-bond accepting A^−^ atom approaches the D_3_Pn molecule (see the Theoretical methods section). The activation strain and energy decomposition diagrams (ASD and EDD) for a representative example series, namely F_3_N⋯F^−^ to F_3_Sb⋯F^−^, are given in Fig. 1 (for the complete dataset, see Tables S1 and S2 in the ESI†). Notably, the trend in bond energy Δ*E*(*ζ*) is in fact determined by Δ*E*_int_(*ζ*), which strengthens when going from Pn = N to Sb (Fig. 1, left), whereas the Δ*E*_strain_(*ζ*) curves are relatively similar. In fact, only in the equilibrium geometries, the strain term Δ*E*_strain_(*ζ*_eq_) become less destabilizing from Pn = N to Sb. The reason is that as the interaction curve becomes steeper along the series, it pulls the equilibrium geometry [which results from the balance between Δ*E*_strain_(*ζ*) and Δ*E*_int_(*ζ*)] to an earlier stage along the reaction coordinate, at which the system is less distorted (*i.e.*, a less expanded F^1^–Pn bond in the F_3_Pn fragment) and thus less strained, as reflected by Δ*E*_strain_(*ζ*_eq_) (see Table 1).

**Fig. 2:**
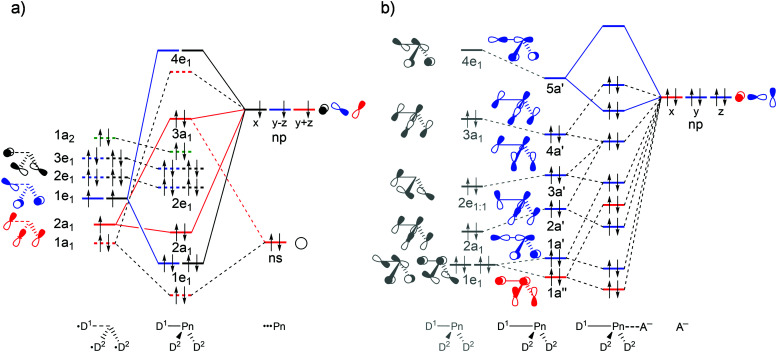
Schematic molecular orbital diagram for (a) isolated D_3_Pn fragments with *C*_3v_ symmetry (red: a_1_; green: a_2_; blue: e_1:1_; black: e_1:2_) and (b) D_3_Pn⋯A^−^ complexes. The first column in (b) refers to the isolated D_3_Pn fragment and the second column refers to the D_3_Pn fragment deformed to its *C*_s_ symmetric geometry in the complex (blue: a′; red: a′′), in which one D–Pn bond has been elongated. See Fig. S1 in the ESI† for computed 3D isosurfaces of the orbitals.

To understand the trends in Δ*E*_int_(*ζ*), we further decomposed Δ*E*_int_ into the individual energy components (Fig. 1, right; for a full set of data, see Fig. S2 in the ESI†). The strengthening of Δ*E*_int_(*ζ*) and, consequently, the increasing stabilization of D_3_Pn⋯A^−^ as Pn varies along N, P, As, and Sb is caused by the rising electronegativity difference across the D–Pn bonds as Pn descends in the periodic table. Firstly, this causes the Pn atom to become increasingly positive along N, P, As, and Sb (see the VDD atomic charges in Table 2), resulting in the Δ*V*_elstat_(*ζ*) curves being the least stabilizing for Pn = N and the most stabilizing for Pn = Sb. For example, the VDD atomic charge on Pn in F_3_N, F_3_P, F_3_As, and F_3_Sb amounts to +0.21, +0.33, +0.51, and +0.57 a.u., respectively. Secondly, this causes, among other effects that will be explained later, the σ* D–Pn antibonding 5a′ acceptor orbital to have higher amplitude on Pn (see Fig. 2), resulting in stronger HOMO–LUMO overlap and thus more stabilizing orbital interactions. These features are also observed for chalcogen bonds D_2_Ch⋯A^−^, halogen bonds DX⋯A^−^, and hydrogen bonds DH⋯A^−^, which makes them similar to pnictogen bonds.^8^

**Table 2:** Bond lengths (in Å), bond angle (in deg.), VDD charge (in a.u.), orbital energies (in eV) and the homolytic bond dissociation energy without ZPE (in kcal mol^−1^) of isolated D_3_Pn fragments^a^

D_3_Pn	*r*_D^1^–Pn_	*r*_D^2^–Pn_	*Θ*_1_	*Θ*_2_	*Q* ^VDD^_Pn_	*ε*(1e_1_)	*ε*(2a_1_)	*ε*(3a_1_)	*ε*(4e_1_)	BDE_D–Pn_^b^
F_3_N	1.356	1.356	101.9	101.9	0.21	−18.2	−16.8	−10.3	0.6	59.4
Cl_3_N	1.753	1.753	107.7	107.7	0.01	−14.4	−13.0	−8.2	−1.3	35.6
Br_3_N	1.906	1.906	108.5	108.5	−0.09	−13.1	−11.8	−7.6	−1.4	30.2
F_3_P	1.564	1.564	97.5	97.5	0.33	−16.1	−15.1	−9.3	−0.5	133.6
Cl_3_P	2.055	2.055	100.4	100.4	0.32	−12.8	−11.7	−8.3	−1.4	79.0
Br_3_P	2.227	2.227	100.9	100.9	0.25	−11.8	−10.8	−7.9	−1.7	60.8
F_3_As	1.717	1.717	95.8	95.8	0.51	−14.7	−13.8	−10.1	−1.3	116.4
Cl_3_As	2.179	2.179	99.1	99.1	0.44	−12.2	−11.2	−8.7	–1.7	76.4
Br_3_As	2.342	2.342	99.7	99.7	0.37	−11.3	−10.4	−8.1	−1.9	59.7
F_3_Sb	1.893	1.893	94.3	94.3	0.57	−13.5	−12.8	−9.7	−1.9	117.9
Cl_3_Sb	2.348	2.348	97.0	97.0	0.55	−11.5	−10.6	−8.5	−2.1	82.8
Br_3_Sb	2.510	2.510	97.7	97.7	0.49	−10.7	−9.9	−8.0	−2.2	66.2

a Computed at the ZORA-M06/QZ4P level.

b Energy for the reaction D_3_Pn → D_2_Pn˙ + D˙.

**Fig. 3:**
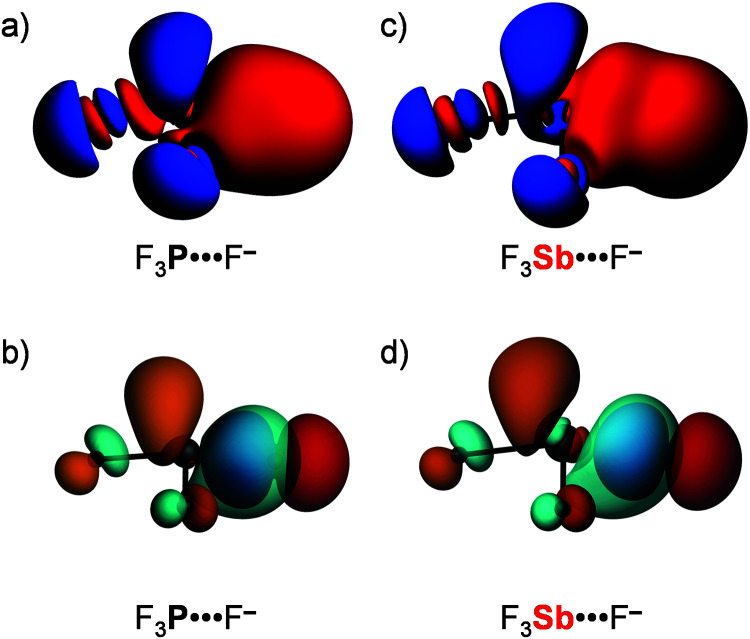
Deformation density (Δ*ρ*(*r*) = *ρ*_[D3Pn⋯A^−^]_(*r*) − *ρ*_D3Pn_(*r*) − *ρ*_A^−^_(*r*); red = depletion; blue = accumulation) plot (a and c) and HOMO–LUMO interaction (b and d) for a representative series of D_3_Pn⋯A^−^ pnictogen bonds.

Our analyses reveal that the pnictogen bonding mechanism is not purely electrostatic but, instead, has a relatively large covalent component (Δ*E*_oi_), stemming mainly from the HOMO–LUMO interaction between the occupied halide *n*p_y_ atomic orbital (AO) and the σ* D–Pn antibonding 5a′ acceptor orbital (see Fig. 2). For the pnictogen-bonded complexes, the orbital-interaction term ranges from 34% for F_3_Sb⋯F^−^ to as much as 65% for Br_3_N⋯Cl^−^ of the total bonding interactions (Δ*E*_oi_ + Δ*V*_elstat_; see Table 3), and the orbital interaction curves Δ*E*_oi_(*ζ*) become more stabilizing from Pn = N to Sb (Fig. 1, right). The stronger orbital interaction for the heavier pnictogens is the result of the larger LUMO–HOMO overlap (*i.e.* 〈5a′|*n*p_y_〉; see Fig. 2 for the MO diagram that depicts the *n*p_y_ orbital of A^−^ oriented towards the D^1^–Pn bond of the D_3_Pn fragment) as Pn becomes more electropositive. For example, in the Cl_3_Pn⋯Cl^−^ series, 〈5a′|*n*p_y_〉 increases from 0.11 to 0.20 to 0.22 along Pn = N, P, and Sb in the equilibrium geometry (see Table 3). The associated charge transfer from A^−^ to D_3_Pn is reflected by the VDD charge of the D_3_Pn fragment in the complex, 
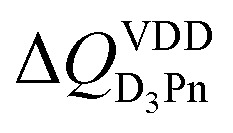
, which is negative (see Table 1). Thus, D_3_Pn gains charge from A^−^ upon complexation, for all D_3_Pn⋯A^−^ complexes. For example, 
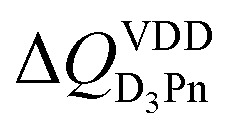
 is −0.30 a.u. for F_3_N⋯F^−^ and −0.31 a.u. for F_3_Sb⋯F^−^. The HOMO–LUMO charge transfer nature of the pnictogen bond is also reflected by the 3D plots of the deformation densities associated with pnictogen-bond formation in F_3_P⋯F^−^ and F_3_Sb⋯F^−^ (see Fig. 3). Note the charge depletion in the region of the HOMO on the Lewis base F^−^ (and within the Pn⋯F^−^ bond due to Pauli repulsion^10*a*^) and the charge accumulation in the region of the LUMO on D_3_Pn.

**Table 3:** Energy decomposition analyses (in kcal mol^−1^) of a representative set of D_3_Pn⋯A^−^ at the equilibrium geometries^*a*^

D_3_Pn⋯A^−^	Δ*E*_int_	Δ*V*_elstat_	Δ*E*_Pauli_	Δ*E*_oi_	*ε*(5a′)	〈5a′|*n*p_y_〉	〈4a′|*n*p_y_〉	Pop_5a′_	Pop_*n*py_	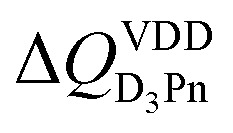
F_3_N⋯F^−^	−44.3	−66.9	89.6	−67.0	−5.1	0.12	0.05	0.40	1.68	−0.30
F_3_N⋯Cl^−^	−4.3	−5.8	4.5	−3.1	–0.2	0.10	0.05	0.02	1.99	−0.01
F_3_N⋯Br^−^	−3.5	−4.7	3.5	−2.3	−0.1	0.10	0.04	0.02	2.00	0.00
Cl_3_N⋯F^−^	−86.1	−208.3	431.6	−309.5	−6.5	0.13	0.08	1.24	1.34	−0.65
Cl_3_N⋯Cl^−^	−28.5	−41.8	69.2	−55.9	−5.2	0.11	0.06	0.54	1.59	−0.36
Cl_3_N⋯Br^−^	−9.7	−11.9	18.8	−16.7	−3.7	0.09	0.03	0.26	1.75	−0.18
Br_3_N⋯F^−^	−83.3	−204.1	439.6	−318.8	−6.0	0.12	0.07	1.25	1.38	−0.67
Br_3_N⋯Cl^−^	−10.0	−10.3	19.2	−18.9	−3.6	0.08	0.04	0.23	1.86	−0.18
Br_3_N⋯Br^−^	−24.2	−51.1	95.0	−68.1	−4.6	0.12	0.08	0.67	1.60	−0.44
F_3_P⋯F^−^	−66.4	−167.7	221.7	−120.3	−2.1	0.15	0.19	0.28	1.75	−0.35
F_3_P⋯Cl^−^	−20.4	−40.4	46.0	−25.9	−1.2	0.22	0.17	0.17	1.87	−0.12
F_3_P⋯Br^−^	−15.9	−29.1	30.7	−17.4	–1.1	0.25	0.16	0.18	1.81	−0.09
Cl_3_P⋯F^−^	–99.3	−222.6	312.1	−188.7	−4.1	0.16	0.13	0.48	1.70	−0.52
Cl_3_P⋯Cl^−^	−39.8	−84.8	119.2	−74.2	−3.1	0.20	0.12	0.40	1.73	−0.31
Cl_3_P⋯Br^−^	−31.3	−64.2	86.5	−53.6	−2.9	0.20	0.08	0.36	1.75	−0.26
Br_3_P⋯F^−^	−101.2	−228.4	331.2	−204.1	−4.2	0.15	0.12	0.53	1.69	−0.56
Br_3_P⋯Cl^−^	−43.9	−95.9	141.9	−89.8	−3.4	0.19	0.13	0.45	1.72	−0.37
Br_3_P⋯Br^−^	−35.3	−73.7	104.8	−66.5	−3.2	0.20	0.12	0.42	1.72	−0.32
F_3_Sb⋯F^−^	−80.8	−148.9	143.6	−75.5	−3.0	0.16	0.15	0.20	1.82	−0.31
F_3_Sb⋯Cl^−^	−44.7	−82.6	83.4	−45.4	−2.7	0.23	0.18	0.21	1.83	−0.21
F_3_Sb⋯Br^−^	−38.7	−70.7	70.9	−38.9	−2.7	0.24	0.18	0.21	1.84	−0.19
Cl_3_Sb⋯F^−^	−89.4	−158.0	161.0	−92.4	−3.4	0.16	0.11	0.26	1.79	−0.38
Cl_3_Sb⋯Cl^−^	−51.7	−93.3	100.9	−59.2	−3.2	0.22	0.11	0.28	1.80	−0.28
Cl_3_Sb⋯Br^−^	−45.3	−81.1	87.3	−51.5	−3.1	0.23	0.10	0.28	1.82	−0.27
Br_3_Sb⋯F^−^	−89.2	−157.4	166.1	−97.9	−3.4	0.16	0.10	0.28	1.79	−0.41
Br_3_Sb⋯Cl^−^	–52.0	−95.2	106.8	−63.5	−3.2	0.21	0.11	0.29	1.81	−0.31
Br_3_Sb⋯Br^−^	−45.8	−83.2	92.7	−55.3	−3.2	0.22	0.11	0.30	1.81	−0.30

a Computed at the ZORA-M06/QZ4P level; *ε*(5a′) = 5a′ orbital energy of the prepared D_3_Pn fragment (in eV); 〈*Φ*|*n*p〉 = overlap between the *Φ* orbital of the D_3_Pn fragment (see Fig. 2) and one of the *n*p orbitals of the halide A^−^; Pop = Gross population (in electrons) of the indicated orbital. For a full set of data, see Table S2 in the ESI.

The Δ*E*_oi_(*ζ*) curves become more stabilizing if one goes from nitrogen to the heavier pnictogen bonds, but, interestingly, the orbital interaction Δ*E*_oi_(*ζ*_eq_) at the stationary point of the complex turns out to be comparable in magnitude for all pnictogens (see Table 3). The reason is the significantly more pronounced stretch in the F^1^–Pn bond for Pn = N than for the heavier pnictogens. This phenomenon causes Δ*E*_oi_(*ζ*_eq_) for nitrogen to occur at a later point at which the intrinsically less stabilizing Δ*E*_oi_(*ζ*) curve has achieved a more stabilizing value that, as mentioned above, is comparable to the value of the other pnictigen bonds that do not feature this strong F^1^–Pn bond stretch.

**Fig. 4:**
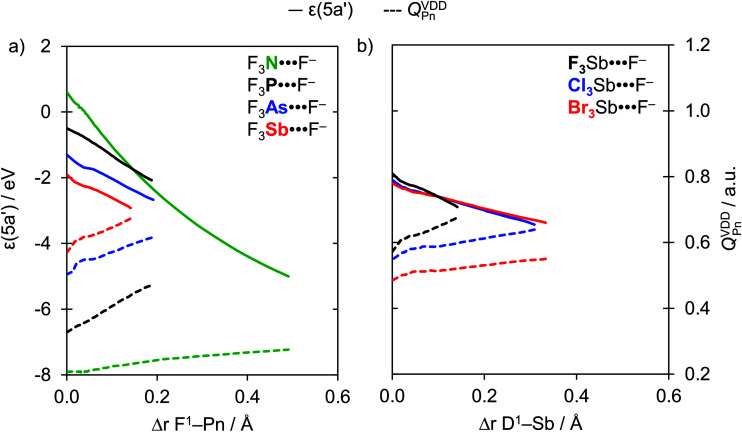
Energy of the 5a′ orbital (in eV) and the VDD charge on the Pn atom (in a.u.) in the neutral fragment D_3_Pn projected onto (a) the F^1^–Pn bond stretch (green, Pn = N; black, Pn = P; blue, Pn = As; red, Pn = Sb) and (b) the D^1^–Sb bond stretch (black, D = F; blue, D = Cl; red, D = Br).

The reason that F^1^–Pn streches more for Pn = N than for the heavier pnictogens is its lower polarity and thus weaker bond strength (see Table 2) which translates into less strain when it streches upon complexation with the Lewis base (see Fig. 1). Thus, as the F^1^–Pn bond in the F_3_Pn⋯F^−^ complexes expands the most, the σ* D–Pn antibonding 5a′ acceptor orbital drops significantly in energy and, due to a smaller HOMO–LUMO gap, enters into a more stabilizing donor–acceptor orbital interaction Δ*E*_oi_(*ζ*_eq_) (see Fig. 2b). This effect can be observed in Fig. 4a, which shows the energies of the σ* F–Pn antibonding 5a′ acceptor orbitals, as well as the VDD atomic charge on Pn in the F_3_Pn fragments, along the reaction coordinate. The F^1^–Pn bond in the F_3_Pn⋯F^−^ complexes expands less from Pn = N to Sb and leads to a smaller stabilization of the σ* D–Pn antibonding 5a′ acceptor orbital and the decrease in the HOMO–LUMO gap becomes smaller from Pn = N to Sb, resulting in less stabilization by orbital interactions. For example, for Pn = Sb, the energy of the σ* F–Sb antibonding 5a′ acceptor orbital is up to −3.0 eV in F_3_Sb at the equilibrium geometry of the F_3_Sb⋯F^−^ complex. Due to the weaker F–N bond compared to F–Sb (*e.g.* BDE_F–N_ = 59.4 kcal mol^−1^ and BDE_F–Sb_ = 117.9 kcal mol^−1^; see Table 2), the F^1^–N bond expands to a higher extent and the σ* F–N antibonding 5a′ acceptor orbital quickly drops to a value of −5.1 eV.

Nevertheless, the nitrogen-bonded complexes remain the weakest as the high stabilization of the σ* F–N antibonding 5a′ acceptor orbital is counteracted by its poor orbital overlap with the *n*p_y_ donor orbital due to the absence of radial nodes in and, thus, the very compact nature of the nitrogen 2p valence AOs (see Table 3).

In addition, note that, as the F^1^–Pn bond in the F_3_Pn⋯F^−^ complexes expands, the pnictogen atom in the D_3_Pn fragment becomes more positive (Fig. 4a), resulting in a more stabilizing electrostatic Δ*V*_elstat_(*ζ*_eq_) (see Table 3). However, the significant expansion of the F^1^–N bond is not enough to make the highly electronegative N atom as positive as the heavier pnictogens, making the nitrogen-bonded complexes also the least stabilized by electrostatic attraction.

### Bond analyses with the variation of A^−^

Our analyses show that the pnictogen bonds D_3_Pn⋯A^−^ become weaker as the Lewis basicity of the A^−^ halide decreases from F^−^ to Br^−^.^17^ Again, this is equivalent to what was found for chalcogen bonds D_2_Ch⋯A^−^, halogen bonds DX⋯A^−^, and hydrogen bonds DH⋯A^−^.^8^ As aforementioned, pnictogen bonds have both an electrostatic component (Δ*V*_elstat_) and a covalent component (Δ*E*_oi_) stemming mainly from the HOMO–LUMO interaction between the occupied halide *n*p atomic orbital (AO) and the σ* D–Pn antibonding 5a′ acceptor orbital, shown schematically in Fig. 2. The electron-donating capacity of the halides is reduced as the halide *n*p AOs become more diffuse and lower in energy from A^−^ = F^−^ to Br^−^, thus weakening Δ*E*_oi_.^8,17*b*^ This will also result in longer bonds and weaker electrostatic attraction. As a result, the interaction energy (Δ*E*_int_) and, thus, the net pnictogen-bond strength Δ*E* become less stabilizing along A^−^ = F^−^ to Br^−^ (see Table 1 and Table S1 in the ESI†).

**Fig. 5:**
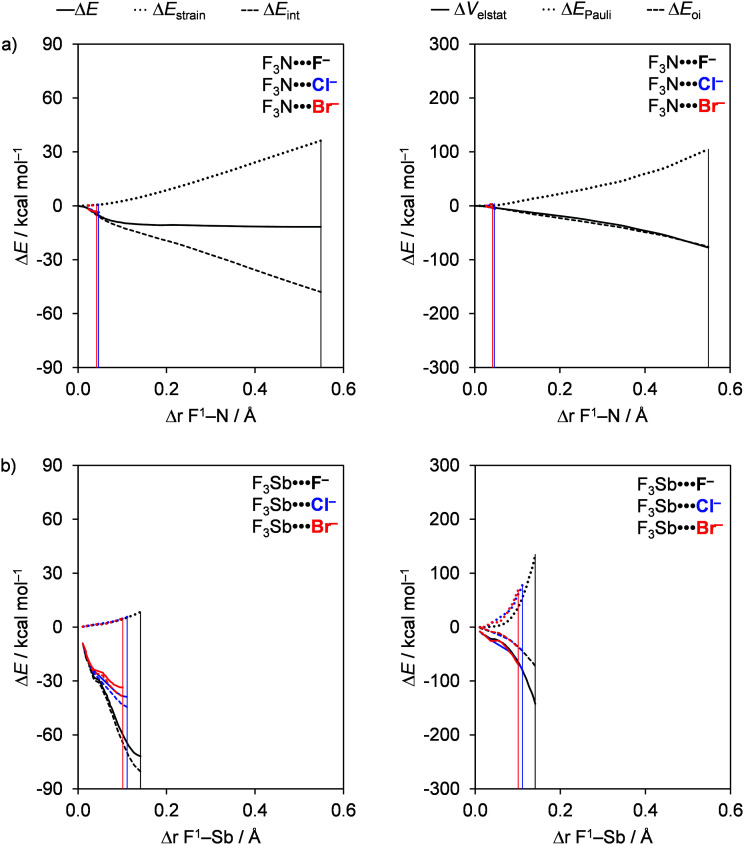
Activation strain (left panel) and energy decomposition (right panel) analyses of (a) F_3_N⋯A^−^ and (b) F_3_Sb⋯A^−^ (black, A^−^ = F^−^; blue, A^−^ = Cl^−^; red, A^−^ = Br^−^). The vertical lines indicate the position of the stationary points.

Activation strain analyses reveal that the trend A^−^ = F^−^ to Br^−^ in the total energy Δ*E*(*ζ*) is directly determined by the trend in the corresponding interaction energies, that is, Δ*E*_int_(*ζ*) weakens from A^−^ = F^−^ to Br^−^. This is nicely seen in the left diagrams in Fig. 5, which shows the formation of the pnictogen bonds D_3_Pn⋯A^−^, with A^−^ = F^−^, Cl^−^, and Br^−^, for a representative series of F_3_N and F_3_Sb molecules. Note that the Δ*E*_strain_ curves coincide (because they stem from the same molecule with the same F–N bond being stretched as the complexation reaction progresses) and, thus, do not affect the trends in Δ*E*(*ζ*). For the nitrogen bonds involving the Lewis bases A^−^ = Cl^−^ and Br^−^, the bond strength is particularly weak because the interaction is too weak to significantly stretch the D^1^–N bonds. Consequently, the D_3_N fragment achieves a much weaker electron-accepting capacity in this less distorted equilibrium geometry and so the eventual interaction and bond energy become relatively weak (see Fig. 5a).

The trend in Δ*E*_int_(*ζ*) is again dictated by the bonding components Δ*V*_elstat_ and Δ*E*_oi_. This can be seen in the right panel of Fig. 5, which shows that both Δ*V*_elstat_(*ζ*) and Δ*E*_oi_(*ζ*) become more stabilizing from A^−^ = F^−^ to Br^−^. The key to understand these trends is of course related to the factors that enhance the strength of the bonding components and, thus, Δ*E*_int_: firstly, an approaching halide A^−^ with a higher lying HOMO and, secondly, a weak D–Pn bond that is easily stretched resulting in a σ* D–Pn antibonding 5a′ acceptor orbital that quickly drops in energy as the D^1^–Pn bond elongates (see Fig. 4). These factors are the driving force for D^1^–Pn stretching in D_3_Pn⋯A^−^ since they generate stronger orbital interactions and, therefore, stronger pnictogen bonds. For F_3_N⋯A^−^, the 5a′ acceptor orbital energy in the equilibrium geometry of the complex adopts significantly lower values for weaker Lewis bases (A^−^ = Cl^−^ or Br^−^) than for A^−^ = F^−^ (*i.e.*, −5.1 eV for A^−^ = F^−^, −0.2 eV for A^−^ = Cl^−^, and −0.1 eV for A^−^ = Br^−^; see Table 3). Indeed, D^1^–Pn stretching is most pronounced if this bond in the neutral fragment is weaker (*e.g.*, *ca.* 59 kcal mol^−1^ for F–N, *ca.* 35 kcal mol^−1^ for Cl–N, and *ca.* 30 kcal mol^−1^ for Br–N; see Table 2). For example, the D^1^–N stretching in the nitrogen-bonded complexes is longer in Br_3_N⋯F^−^, for which Δ*r*_D^1^–Pn_ is 1.4 Å, and less pronounced in F_3_N⋯F^−^, for which Δ*r*_D^1^–Pn_ is 0.5 Å (see Table 1). As a result, the bonding components, Δ*V*_elstat_ and Δ*E*_oi_, become significantly stronger, up to −300 kcal mol^−1^, in the Cl_3_N⋯A^−^ and the Br_3_N⋯A^−^ series. However, the bonding components are significantly weakened to *ca.* −60 kcal mol^−1^ for weaker Lewis bases, in which case Δ*r*_D^1^–Pn_ varies between only 0.04 and 0.6 Å (see Table 1).

### Bond analyses with the variation of D

**Fig. 6:**
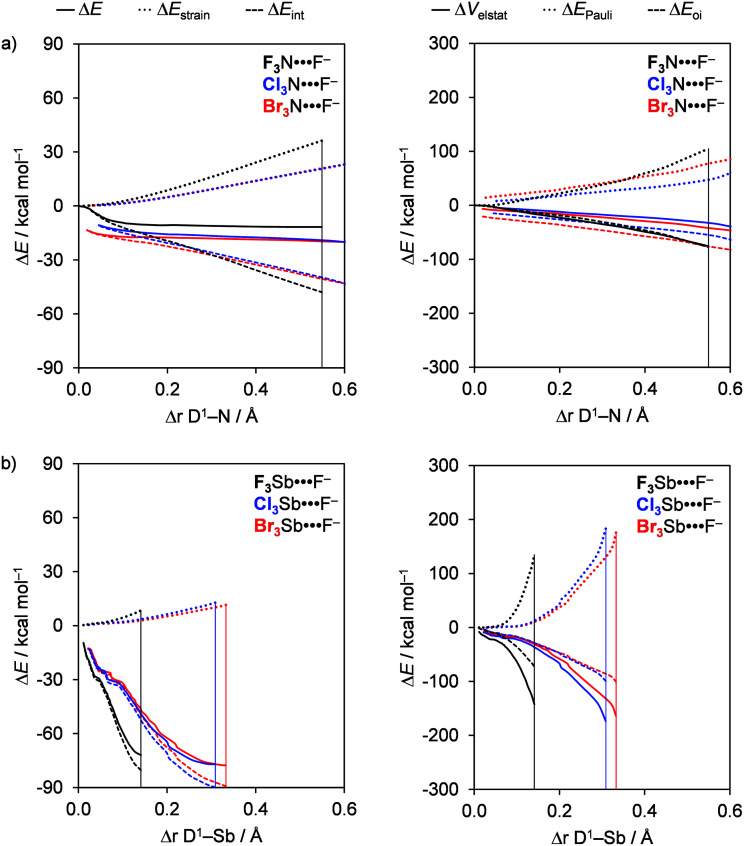
Activation strain (left panel) and energy decomposition (right panel) analyses of (a) D_3_N⋯F^−^ and (b) D_3_Sb⋯F^−^ (black, D = F; blue, D = Cl; red, D = Br). The vertical lines indicate the position of the stationary points.

The strength of the heavier D_3_Pn⋯A^−^ pnictogen bonds (Pn = P, As, Sb) slightly strengthens when the substituent D varies from F to Br. Based on the purely electrostatic picture of the σ-hole model, one might expect just the opposite, that is, a weakening of the pnictogen bond in D_3_Pn⋯A^−^ as D varies from F to Br due to a decrease in the positive molecular electrostatic potential of the σ-hole at the Pn atom in the D_3_Pn fragment (*V*_S,max_).^6*a*,18^ This apparent discrepancy from the σ-hole model is traced to the trend that D–Pn bonds become weaker along F–Pn, Cl–Pn and Br–Pn (see Table 2). And, the weaker the D–Pn bonds, the more they elongate in the eventual equilibrium geometry of the corresponding D_3_Pn⋯A^−^ complex. Therefore, the latter is reached at a later stage along the reaction coordinate Δ*r*_D^1^–Pn_. A consequence of this D–Pn bond elongation is a more electropositive Pn atom and a lower energy of the σ* D–Pn antibonding 5a′ acceptor orbital due to a reduction in antibonding character (see Fig. 4b). This situation translates into more stabilizing Δ*V*_elstat_(*ζ*) and Δ*E*_oi_(*ζ*) curves as D varies from F to Cl to Br (see Fig. 6, right). For the D_3_N⋯A^−^ nitrogen bonds, these effects are most pronounced because the D–N bonds in D_3_N are the weakest halogen–pnictogen bonds. Thus, stronger D_3_N⋯A^−^ complexes with a more pronounced D–N stretch occur if we go from F_3_N to Cl_3_N and Br_3_N.

In the following, we exemplify the above with a few concrete examples. For the heavier pnictogen bonds (Pn = P, As, Sb), we find that the trend in bond energy curves Δ*E*(*ζ*) is set by the interaction energy curves Δ*E*_int_(*ζ*), that is, they become less stabilizing along D = F, Cl, and Br. However, as the D^1^–Pn bond expands more for D = Cl and Br compared to D = F, both Δ*E*(*ζ*_eq_) and Δ*E*_int_(*ζ*_eq_) in the equilibrium geometry are slightly more stabilizing for D = Cl and Br. This trend in the interaction energy curves Δ*E*_int_(*ζ*) is a direct consequence of the electrostatic attraction Δ*V*_elstat_ and orbital interactions Δ*E*_oi_. The curves for the bonding components Δ*V*_elstat_(*ζ*) and Δ*E*_oi_(*ζ*) are the most stabilizing for D = F because of the larger difference in electronegativity across the D–Pn bonds (*vide supra*). Nevertheless, the Cl–Pn and Br–Pn bonds are substantially weaker than the associated F–Pn bond (*e.g.* BDE_F–Sb_ = 117.9 kcal mol^−1^, BDE_Cl–Sb_ = 82.8 kcal mol^−1^, and BDE_Br–Sb_ = 66.2 kcal mol^−1^; see Table 2), and the Cl–Pn and Br–Pn bonds expand to a greater degree. Consequently, Δ*E*_oi_(*ζ*) becomes more stabilizing for D = Cl and Br because the σ* D–Pn antibonding 5a′ acceptor orbital is strongly stabilized and can engage in stronger donor–acceptor interactions with the *n*p-type lone pair HOMO on A^−^ (see Fig. 4b). In parallel, the VDD atomic charge on Pn becomes increasingly more positive as the D^1^–Pn bond expands (see Fig. 4b), which translates into a more stabilizing Δ*V*_elstat_(*ζ*) for D = Cl and Br. This results in the slight strengthening of Δ*V*_elstat_(*ζ*_eq_) and Δ*E*_oi_(*ζ*_eq_), and thus Δ*E*_int_(*ζ*_eq_), in the equilibrium geometry along D = F, Cl, Br.

The D_3_N⋯A^−^ complexes, on the other hand, show somewhat deviating behavior as compared to the heavier pnictogen bonding complexes exemplified above. Thus, for the D_3_N⋯A^−^ complexes, Δ*E*(*ζ*) becomes significantly more stabilizing along D = F, Cl, and Br instead of remaining relatively constant. This is because the D^1^–N bonds are all much weaker (*e.g.* BDE_F–N_ = 59.4 kcal mol^−1^, BDE_Cl–N_ = 35.6 kcal mol^−1^, and BDE_Br–N_ = 30.2 kcal mol^−1^; see Table 2), and the stretch Δ*r*_D^1^–Pn_ for all nitrogen-bonded complexes is much more pronounced, that is, the complexes occur later in the reaction coordinate. For example, the D^1^–N stretch has variation between 0.5 and 1.4 Å in D_3_N⋯F^−^, whereas the D^1^–Sb stretch varies only between 0.1 and 0.3 Å in D_3_Sb⋯F^−^ from D = F to Br (see Table 1). As a result, the D_3_N⋯A^−^ complexes show more significant strengthening of Δ*V*_elstat_(*ζ*_eq_) and Δ*E*_oi_(*ζ*_eq_) along D = F to Br in the equilibrium geometries, and, therefore, a significant increase in stability along the same series.

### Comparison of pnictogen-, chalcogen-, halogen-, and hydrogen bonds

**Fig. 7:**
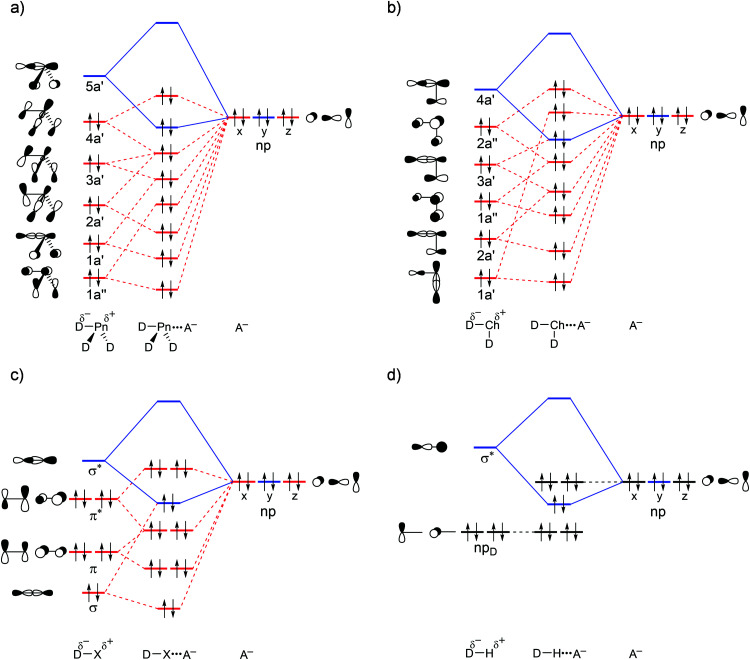
Generic molecular orbital diagrams for (a) D_3_Pn⋯A^−^ pnictogen bonds, (b) D_2_Ch⋯A^−^ chalcogen bonds, (c) DX⋯A^−^ halogen bonds, and (d) DH⋯A^−^ hydrogen bonds.

Our analyses highlight that pnictogen bonds share strong similarities with the corresponding chalcogen bonds (ChB), halogen bonds (XB), and hydrogen bonds (HB).^8^ We find that these bonds have considerable covalency on top of electrostatic attraction and can range in strength roughly between −3 and −78 kcal mol^−1^ (see Fig. 7). The contribution of the covalent component Δ*E*_oi_ to the total bonding components (Δ*V*_elstat_ + Δ*E*_oi_) is up to 97%, 76%, and 65% for XB, ChB, and PnB, respectively, whereas it is up to 66% for HB.^8*a*,*b*^ The same bonding mechanism with a substantial covalent component is also observed for the archetypal DM⋯A^−^ alkali- and coinage-metal bonds (MB) that have even more pronounced polarization in the D–M bonds.^8*c*^

Our findings consolidate earlier work and support the charge-transfer character of pnictogen bonds by providing a causal bonding mechanism.^2*b*,7^ PnB, ChB, and XB are generally stronger than HB due to more stabilizing orbital interactions (see Table S3 for bond energies Δ*E* of a representative series of ChB, XB, and HB). On the other hand, hydrogen bonds have less destabilizing Pauli repulsion because there is no overlap between the np_D_ HOMO of the D–H fragment and the higher-lying occupied np AOs of A^−^ (see Fig. 7). Our analyses also show that PnB, ChB, and XB, but also MB, have even stronger electrostatic attraction than HB.^8*a*,*b*^ Note that this cannot be straightforwardly explained by the σ-hole model, which, based on hydrogen having the highest *V*_S,max_, erroneously suggests that HB should have the stronger electrostatic attraction.^6^

## Conclusions

The pnictogen bonds in D_3_Pn⋯A^−^ range between 3 and 78 kcal mol^−1^ in strength, becoming stronger as the pnictogen atom becomes more electropositive, along Pn = N, P, As and Sb, and also as the halide becomes a stronger Lewis base, along A^−^ = Br^−^, Cl^−^ and F^−^. The trend upon variation of the substituent along D = F, Cl, Br is less pronounced, as are all trends for the relatively weak nitrogen bonds. This follows from our bonding analyses based on relativistic density functional theory.

Our activation-strain and quantitative Kohn–Sham MO bonding analyses reveal that the pnictogen bonds in D_3_Pn⋯A^−^ have a considerable covalent component Δ*E*_oi_, ranging from 34% to 65% of the bonding components (Δ*V*_elstat_ + Δ*E*_oi_), stemming from HOMO–LUMO interactions between the *n*p-type lone pair HOMO on A^−^ and the σ* D–Pn antibonding LUMO on D_3_Pn. The D_3_Pn⋯A^−^ pnictogen bond becomes stronger as Pn descends in the periodic table along N, P, As and Sb. One reason is the increasing polarization towards Pn of the σ* LUMO and the associated increase in the LUMO–HOMO overlap with A^−^ (along P, As, and Sb this trend is reinforced by the drop in the σ* LUMO energy). Another reason is the higher positive charge on Pn which goes with more stabilizing electrostatic interactions with the Lewis base.

Finally, it appears that the pnictogen bonds in D_3_Pn⋯A^−^ are similar in nature to the chalcogen bonds in D_2_Ch⋯A^−^, halogen bonds in DX⋯A^−^, and hydrogen bonds in DH⋯A^−^ (Pn = N, P, As, Sb; Ch = O, S, Se, Te; D, X, A = F, Cl, Br). Our work constitutes a unified picture of all these interactions, which appear to be far from solely electrostatic phenomena. We conclude that the often-used designation of “noncovalent interactions” for these types of bonds is not consistent with their significant covalent nature. Instead of this term, we propose to refer to such bonds as (weak or strong) intermolecular interactions.

## Conflicts of interest

There are no conflicts to declare.

## Supplementary Material

CP-023-D1CP01571K-s001
