# Supplementary material for: The pnictogen bond: a quantitative molecular orbital picture
Source: Phys Chem Chem Phys. 2021 Jun 4;23(25):13842–52. doi: 10.1039/d1cp01571k (PMC8297534; doi:10.1039/d1cp01571k)
Supplement: CP-023-D1CP01571K-s001 [file CP-023-D1CP01571K-s001.pdf]

Contents

Fig. S1. Schematic FMO and DFT MO isosurfaces (at 0.04 a.u.) for a) the $(D^{\bullet})_3$ molecule in its quadruplet valence state at C_{3v} symmetry and for the D_3Pn fragment in b) the ground state at C_{2v} symmetry and in c) the geometry of the complex at C_s symmetry.

Fig. S2. Activation strain (left panel) and energy decomposition (right panel) analyses of $F_3Pn{\bullet}{\bullet}{\bullet}F^-$ pnictogen-bonded complexes (green, Pn = N; black, Pn = P; blue, Pn = As; red, Pn = Sb). The vertical lines indicate the position of the stationary points.

Table S1. Activation strain analyses (in kcal mol^{-1}) of $D_3Pn{\bullet}{\bullet}{\bullet}A^-$ pnictogen–bonds at the equilibrium geometries (in Å, deg.).

Table S2. Energy decomposition analyses (in kcal mol^{-1}) of $D_3Pn{\bullet}{\bullet}{\bullet}A^-$ pnictogen–bonds at the equilibrium geometries.

Table S3. Activation strain and energy decomposition analyses (in kcal mol^{-1}) of $D_mZ{\bullet}{\bullet}{\bullet}A^-$ hydrogen bonds, halogen bonds, chalcogen bonds, and pnictogen bonds at the equilibrium geometries.

Table S4. Cartesian coordinates, bonding energies (in kcal mol^{-1}) and the number of imaginary vibrational frequencies (N_{imag}) for all stationary points of $D_2N^{\bullet}$, D_3N and $D_3N{\bullet}{\bullet}{\bullet}A^-$ structures, computed at ZORA-M06/QZ4P.

Table S5. Cartesian coordinates, bonding energies (in kcal mol^{-1}) and the number of imaginary vibrational frequencies (N_{imag}) for all stationary points of $D_2P^{\bullet}$, D_3P and $D_3P{\bullet}{\bullet}{\bullet}A^-$ structures, computed at ZORA-M06/QZ4P.

Table S6. Cartesian coordinates, bonding energies (in kcal mol^{-1}) and the number of imaginary vibrational frequencies (N_{imag}) for all stationary points of $D_2As^{\bullet}$, D_3As and $D_3As{\bullet}{\bullet}{\bullet}A^-$ structures, computed at ZORA-M06/QZ4P.

Table S7. Cartesian coordinates, bonding energies (in kcal mol^{-1}) and the number of imaginary vibrational frequencies (N_{imag}) for all stationary points of $D_2Sb^{\bullet}$, D_3Sb and $D_3Sb{\bullet}{\bullet}{\bullet}A^-$ structures, computed at ZORA-M06/QZ4P.

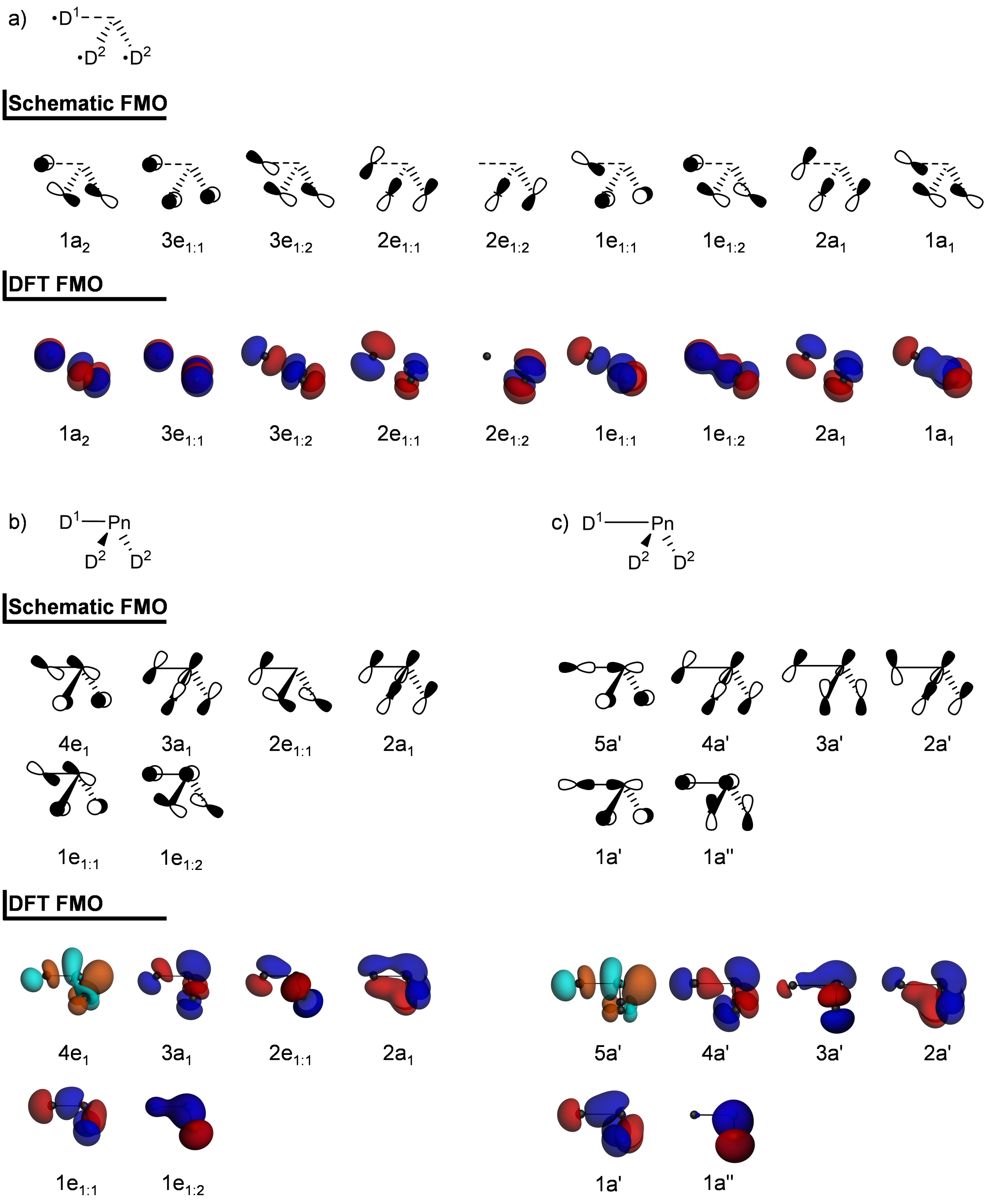

Fig. S1. Schematic FMO and DFT MO isosurfaces (at 0.04 a.u.) for (a) the $(D^{\bullet})_3$ molecule in its quadruplet valence state at C_{3v} symmetry and for the D_3Pn fragment in (b) the ground state at C_{2v} symmetry and in (c) the geometry of the complex at C_s symmetry.

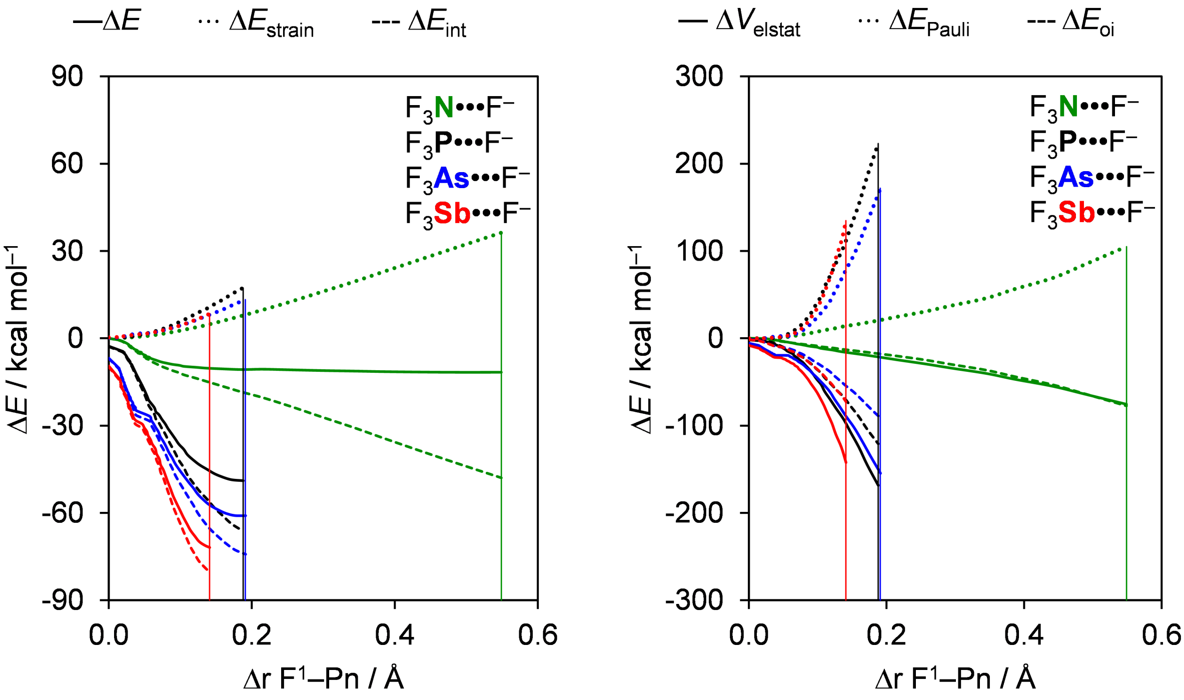

Fig. S2. Activation strain (left panel) and energy decomposition (right panel) analyses of $F_3Pn\cdots F^-$ pnictogen-bonded complexes (green, Pn = N; black, Pn = P; blue, Pn = As; red, Pn = Sb). The vertical lines indicate the position of the stationary points.

Table S1. Activation strain analyses (in kcal mol^{-1}) of $D_3Pn\cdots A^-$ pnictogen bonds at the equilibrium geometries (in Å, deg.).[a]

$D_3Pn\cdots A^-$	ΔE	ΔE_{strain}	ΔE_{int}	$r_{Pn\cdots A^-}$	r_{Pn-D^1}	r_{Pn-D^2}	Δr_{Pn-D^1}	Δr_{Pn-D^2}	Θ_1	Θ_2	Θ_3	$\Delta\Theta_1$	$\Delta\Theta_2$
$F_3N\cdots F^-$	–11.8	32.5	–44.3	1.859	1.859	1.339	0.503	–0.017	93.0	102.4	170.3	–8.9	0.5
$F_3N\cdots Cl^-$	–3.5	0.8	–4.3	3.239	1.404	1.347	0.048	–0.009	100.4	102.3	168.3	–1.5	0.4
$F_3N\cdots Br^-$	–2.9	0.5	–3.5	3.484	1.396	1.348	0.040	–0.008	100.7	102.2	166.8	–1.2	0.3
$Cl_3N\cdots F^-$	–30.4	55.7	–86.1	1.416	3.243	1.754	1.490	0.001	82.0	108.1	170.7	–25.7	0.4
$Cl_3N\cdots Cl^-$	–5.6	22.9	–28.5	2.328	2.328	1.757	0.575	0.004	99.9	106.9	146.6	–7.8	–0.8
$Cl_3N\cdots Br^-$	–6.2	3.5	–9.7	2.920	1.902	1.777	0.149	0.024	104.9	106.8	145.6	–2.8	–0.9
$Br_3N\cdots F^-$	–30.2	53.2	–83.3	1.411	3.323	1.928	1.417	0.022	84.5	108.5	165.7	–24.0	0.0
$Br_3N\cdots Cl^-$	–8.0	2.0	–10.0	2.813	2.017	1.932	0.111	0.026	105.7	107.7	149.4	–2.8	–0.8
$Br_3N\cdots Br^-$	–7.0	17.2	–24.2	2.322	2.322	1.972	0.416	0.066	105.4	106.8	127.2	–3.1	–1.7
$F_3P\cdots F^-$	–48.9	17.4	–66.4	1.753	1.753	1.608	0.189	0.044	86.9	98.7	189.4	–10.6	1.2
$F_3P\cdots Cl^-$	–16.0	4.4	–20.4	2.700	1.647	1.580	0.083	0.016	91.9	97.8	183.4	–5.6	0.3
$F_3P\cdots Br^-$	–12.9	3.0	–15.9	2.982	1.631	1.576	0.067	0.012	92.9	97.7	181.6	–4.6	0.2
$Cl_3P\cdots F^-$	–67.4	31.8	–99.3	1.649	2.627	2.103	0.572	0.048	88.6	100.2	181.4	–11.8	–0.2
$Cl_3P\cdots Cl^-$	–25.5	14.4	–39.8	2.370	2.370	2.089	0.315	0.034	91.7	100.1	174.6	–8.7	–0.3
$Cl_3P\cdots Br^-$	–20.5	10.7	–31.3	2.617	2.318	2.085	0.263	0.030	92.8	100.1	172.9	–7.6	–0.3
$Br_3P\cdots F^-$	–71.0	30.2	–101.2	1.637	2.874	2.272	0.647	0.045	89.6	100.3	175.4	–11.3	–0.6
$Br_3P\cdots Cl^-$	–28.5	15.4	–43.9	2.312	2.609	2.265	0.382	0.038	92.0	100.2	172.0	–8.9	–0.7
$Br_3P\cdots Br^-$	–23.4	11.9	–35.3	2.550	2.550	2.261	0.323	0.034	93.0	100.2	170.6	–7.9	–0.7
$F_3As\cdots F^-$	–61.0	12.6	–73.6	1.900	1.900	1.757	0.183	0.040	87.1	97.2	188.7	–8.7	1.4
$F_3As\cdots Cl^-$	–28.7	6.1	–34.9	2.595	1.836	1.744	0.119	0.027	89.5	95.8	182.3	–6.3	0.0
$F_3As\cdots Br^-$	–24.2	4.8	–29.0	2.820	1.820	1.741	0.103	0.024	90.2	95.6	180.4	–5.6	–0.2
$Cl_3As\cdots F^-$	–70.0	18.7	–88.7	1.850	2.585	2.229	0.406	0.050	91.6	98.7	175.7	–7.5	–0.4
$Cl_3As\cdots Cl^-$	–35.1	11.3	–46.4	2.465	2.465	2.223	0.286	0.044	93.4	98.2	169.7	–5.7	–0.9
$Cl_3As\cdots Br^-$	–30.1	9.6	–39.7	2.668	2.437	2.220	0.258	0.041	93.9	98.2	167.9	–5.2	–0.9
$Br_3As\cdots F^-$	–71.5	17.3	–88.8	1.843	2.789	2.391	0.447	0.049	93.0	99.1	172.0	–6.7	–0.6
$Br_3As\cdots Cl^-$	–37.0	10.7	–47.6	2.438	2.663	2.389	0.321	0.047	94.7	98.6	165.7	–5.0	–1.1
$Br_3As\cdots Br^-$	–32.0	9.2	–41.2	2.634	2.634	2.387	0.292	0.045	95.2	98.6	164.0	–4.5	–1.1
$F_3Sb\cdots F^-$	–72.0	8.8	–80.8	2.037	2.037	1.930	0.144	0.037	85.4	97.0	193.9	–8.9	2.7
$F_3Sb\cdots Cl^-$	–38.9	5.8	–44.7	2.643	2.005	1.923	0.112	0.030	87.0	95.1	188.3	–7.3	0.8
$F_3Sb\cdots Br^-$	–33.6	5.0	–38.7	2.840	1.996	1.921	0.103	0.028	87.5	94.8	186.7	–6.8	0.5
$Cl_3Sb\cdots F^-$	–77.1	12.3	–89.4	2.017	2.649	2.399	0.301	0.051	89.4	97.6	183.7	–7.6	0.6
$Cl_3Sb\cdots Cl^-$	–42.7	8.9	–51.7	2.592	2.592	2.393	0.244	0.045	90.5	96.7	178.4	–6.5	–0.3
$Cl_3Sb\cdots Br^-$	–37.3	8.0	–45.3	2.780	2.577	2.391	0.229	0.043	90.9	96.6	177.0	–6.1	–0.4
$Br_3Sb\cdots F^-$	–77.7	11.5	–89.2	2.014	2.842	2.559	0.332	0.049	90.5	98.0	181.2	–7.2	0.3
$Br_3Sb\cdots Cl^-$	–43.5	8.5	–52.0	2.580	2.782	2.556	0.272	0.046	91.6	97.2	175.6	–6.1	–0.5
$Br_3Sb\cdots Br^-$	–38.1	7.7	–45.8	2.766	2.766	2.555	0.256	0.045	91.9	97.0	174.2	–5.8	–0.7

[a] Computed at ZORA-M06/QZ4P.

Table S2. Energy decomposition analyses (in kcal mol^{-1}) of $D_3Pn{\cdots}A^-$ pnictogen bonds at the equilibrium geometries.[a]

$D_3Pn{\cdots}A^-$	ΔE_{int}	ΔV_{elstat}	ΔE_{Pauli}	ΔE_{oi}	ε(5a')	⟨5a'|np_y⟩	⟨5a'|np_y⟩^{2}	⟨4a'|np_y⟩	$Pop_{5a'}$	Pop_{npy}	$\Delta Q_{D_3Pn}^{VDD}$
$F_3N{\cdots}F^-$	–44.3	–66.9	89.6	–67.0	–5.1	0.12	0.01	0.05	0.40	1.68	–0.30
$F_3N{\cdots}Cl^-$	–4.3	–5.8	4.5	–3.1	–0.2	0.10	0.01	0.05	0.02	1.99	–0.01
$F_3N{\cdots}Br^-$	–3.5	–4.7	3.5	–2.3	–0.1	0.10	0.01	0.04	0.02	2.00	0.00
$Cl_3N{\cdots}F^-$	–86.1	–208.3	431.6	–309.5	–6.5	0.13	0.02	0.08	1.24	1.34	–0.65
$Cl_3N{\cdots}Cl^-$	–28.5	–41.8	69.2	–55.9	–5.2	0.11	0.01	0.06	0.54	1.59	–0.36
$Cl_3N{\cdots}Br^-$	–9.7	–11.9	18.8	–16.7	–3.7	0.09	0.01	0.03	0.26	1.75	–0.18
$Br_3N{\cdots}F^-$	–83.3	–204.1	439.6	–318.8	–6.0	0.12	0.01	0.07	1.25	1.38	–0.67
$Br_3N{\cdots}Cl^-$	–10.0	–10.3	19.2	–18.9	–3.6	0.08	0.01	0.04	0.23	1.86	–0.18
$Br_3N{\cdots}Br^-$	–24.2	–51.1	95.0	–68.1	–4.6	0.12	0.01	0.08	0.67	1.60	–0.44
$F_3P{\cdots}F^-$	–66.4	–167.7	221.7	–120.3	–2.1	0.15	0.02	0.19	0.28	1.75	–0.35
$F_3P{\cdots}Cl^-$	–20.4	–40.4	46.0	–25.9	–1.2	0.22	0.05	0.17	0.17	1.87	–0.12
$F_3P{\cdots}Br^-$	–15.9	–29.1	30.7	–17.4	–1.1	0.25	0.06	0.16	0.18	1.81	–0.09
$Cl_3P{\cdots}F^-$	–99.3	–222.6	312.1	–188.7	–4.1	0.16	0.03	0.13	0.48	1.70	–0.52
$Cl_3P{\cdots}Cl^-$	–39.8	–84.8	119.2	–74.2	–3.1	0.20	0.04	0.12	0.40	1.73	–0.31
$Cl_3P{\cdots}Br^-$	–31.3	–64.2	86.5	–53.6	–2.9	0.20	0.04	0.08	0.36	1.75	–0.26
$Br_3P{\cdots}F^-$	–101.2	–228.4	331.2	–204.1	–4.2	0.15	0.02	0.12	0.53	1.69	–0.56
$Br_3P{\cdots}Cl^-$	–43.9	–95.9	141.9	–89.8	–3.4	0.19	0.04	0.13	0.45	1.72	–0.37
$Br_3P{\cdots}Br^-$	–35.3	–73.7	104.8	–66.5	–3.2	0.20	0.04	0.12	0.42	1.72	–0.32
$F_3As{\cdots}F^-$	–73.6	–145.4	158.0	–86.2	–2.6	0.15	0.02	0.15	0.23	1.77	–0.30
$F_3As{\cdots}Cl^-$	–34.9	–64.4	67.6	–38.1	–2.2	0.21	0.04	0.16	0.20	1.84	–0.17
$F_3As{\cdots}Br^-$	–29.0	–52.1	52.8	–29.7	–2.1	0.22	0.05	0.16	0.18	1.85	–0.14
$Cl_3As{\cdots}F^-$	–88.7	–166.5	194.3	–116.5	–3.7	0.16	0.03	0.11	0.34	1.74	–0.42
$Cl_3As{\cdots}Cl^-$	–46.4	–85.5	101.9	–62.8	–3.2	0.21	0.04	0.11	0.33	1.76	–0.29
$Cl_3As{\cdots}Br^-$	–39.7	–71.4	83.2	–51.5	–3.1	0.22	0.05	0.10	0.31	1.78	–0.26
$Br_3As{\cdots}F^-$	–88.8	–167.8	203.9	–124.9	–3.7	0.15	0.02	0.11	0.37	1.75	–0.45
$Br_3As{\cdots}Cl^-$	–47.6	–89.9	112.3	–70.1	–3.3	0.20	0.04	0.11	0.35	1.78	–0.33
$Br_3As{\cdots}Br^-$	–41.2	–75.9	92.9	–58.3	–3.3	0.21	0.04	0.10	0.34	1.76	–0.31
$F_3Sb{\cdots}F^-$	–80.8	–148.9	143.6	–75.5	–3.0	0.16	0.03	0.15	0.20	1.82	–0.31
$F_3Sb{\cdots}Cl^-$	–44.7	–82.6	83.4	–45.4	–2.7	0.23	0.05	0.18	0.21	1.83	–0.21
$F_3Sb{\cdots}Br^-$	–38.7	–70.7	70.9	–38.9	–2.7	0.24	0.06	0.18	0.21	1.84	–0.19
$Cl_3Sb{\cdots}F^-$	–89.4	–158.0	161.0	–92.4	–3.4	0.16	0.03	0.11	0.26	1.79	–0.38
$Cl_3Sb{\cdots}Cl^-$	–51.7	–93.3	100.9	–59.2	–3.2	0.22	0.05	0.11	0.28	1.80	–0.28
$Cl_3Sb{\cdots}Br^-$	–45.3	–81.1	87.3	–51.5	–3.1	0.23	0.05	0.10	0.28	1.82	–0.27
$Br_3Sb{\cdots}F^-$	–89.2	–157.4	166.1	–97.9	–3.4	0.16	0.03	0.10	0.28	1.79	–0.41
$Br_3Sb{\cdots}Cl^-$	–52.0	–95.2	106.8	–63.5	–3.2	0.21	0.04	0.11	0.29	1.81	–0.31
$Br_3Sb{\cdots}Br^-$	–45.8	–83.2	92.7	–55.3	–3.2	0.22	0.05	0.11	0.30	1.81	–0.30

[a] Computed at ZORA-M06/QZ4P; ε(5a') = 5a' orbital energy of the prepared D_3Pn fragment (in eV); ⟨Φ\|np⟩ = overlap between the Φ orbital of the D_3Pn fragment (see Fig. 2) and one of the np orbitals of the halide A^-; Pop = Gross population (in electrons) of indicated orbital.

Table S3. Activation strain and energy decomposition analyses (in kcal mol^{-1}) of $D_mZ{\cdots}A^-$ hydrogen bonds, halogen bonds, chalcogen bonds, and pnictogen bonds at the equilibrium geometries.[a]

$D_mZ{\cdots}A^-$	ΔE	ΔE_{strain}	ΔE_{int}	ΔV_{elstat}	ΔE_{Pauli}	ΔE_{oi}
**$FH{\cdots}F^-$** [b]	–45.8	22.1	–67.9	–76.2	72.9	–64.6
**$FF{\cdots}F^-$** [b]	–30.5	28.0	–58.5	–44.6	84.3	–98.1
**$FCl{\cdots}F^-$** [b]	–52.3	14.0	–66.4	–94.5	124.9	–96.7
**$FBr{\cdots}F^-$** [b]	–62.3	10.0	–72.3	–104.0	113.8	–82.0
**$FI{\cdots}F^-$** [b]	–70.3	6.9	–77.2	–116.9	115.3	–75.7
**$F_2O{\cdots}F^-$** [b]	–21.9	28.3	–50.2	–55.3	87.5	–82.4
**$F_2S{\cdots}F^-$** [b]	–50.1	16.2	–66.3	–126.5	168.3	–108.1
**$F_2Se{\cdots}F^-$** [b]	–62.4	11.4	–73.7	–124.6	136.6	–85.7
**$F_2Te{\cdots}F^-$** [b]	–72.4	7.9	–80.3	–134.6	132.8	–78.6
**$F_3N{\cdots}F^-$**	–11.8	32.5	–44.3	–66.9	89.6	–67.0
**$F_3P{\cdots}F^-$**	–48.9	17.4	–66.4	–167.7	221.7	–120.3
**$F_3As{\cdots}F^-$**	–61.0	12.6	–73.6	–145.4	158.0	–86.2
**$F_3Sb{\cdots}F^-$**	–72.0	8.8	–80.8	–148.9	143.6	–75.5

[a] Computed at ZORA-M06/QZ4P.

[b] Computed at ZORA-M06/QZ4P (from: L. de Azevedo Santos, T. A. Hamlin, T. C. Ramalho, F. M. Bickelhaupt, *ChemistryOpen*, 2021, **10**, 391–401).

Table S4. Cartesian coordinates, bonding energies (in kcal mol^{-1}) and the number of imaginary vibrational frequencies (N_{imag}) for all stationary points of $D_2N^{\cdot}$, D_3N and $D_3N\cdots A^-$ structures, computed at ZORA-M06/QZ4P.

$F_2N^{\cdot}$
E = -411.44
N_{imag} = 0

N	0.000000	0.000000	0.282592
F	0.000000	1.045626	-0.542918
F	0.000000	-1.045626	-0.542918

$Cl_2N^{\cdot}$
E = -319.10
N_{imag} = 0

N	0.000000	0.000000	0.205507
Cl	0.000000	1.390469	-0.754532
Cl	0.000000	-1.390469	-0.754532

$Br_2N^{\cdot}$
E = -297.35
N_{imag} = 0

N	0.000000	0.000000	0.192269
Br	0.000000	1.524174	-0.839361
Br	0.000000	-1.524174	-0.839361

F_3N
E = -534.67
N_{imag} = 0

N	0.000000	0.000000	0.431961
F	-0.608092	1.053247	-0.167194
F	-0.608092	-1.053247	-0.167194
F	1.216184	0.000000	-0.167194

Cl_3N
E = -388.25
N_{imag} = 0

N	0.000000	0.000000	0.436388
Cl	-0.817370	1.415727	-0.197496
Cl	-0.817370	-1.415727	-0.197496
Cl	1.634740	0.000000	-0.197496

Br_3N
E = -361.45
N_{imag} = 0

N	0.000000	0.000000	0.447775
Br	-0.892970	1.546669	-0.217524
Br	-0.892970	-1.546669	-0.217524
Br	1.785940	0.000000	-0.217524

$F_3N\cdots F^-$
E = -683.28
N_{imag} = 0

N	0.268143	-0.004918	0.000000
F	0.361175	1.851683	0.000000
F	-0.570517	-0.034029	1.043030
F	-0.570517	-0.034029	-1.043030
F	0.489462	-1.850792	0.000000

$Cl_3N\cdots F^-$
E = -555.49
N_{imag} = 0

N	0.269408	-0.613135	0.000000
Cl	0.696175	2.601343	0.000000
Cl	-0.689932	-0.238751	1.420412
Cl	-0.689932	-0.238751	-1.420412
F	0.311517	-2.028706	0.000000

$Br_3N\cdots F^-$
E = -528.42
N_{imag} = 0

N	0.308263	-0.668113	0.000000
Br	0.753228	2.625398	0.000000
Br	-0.767698	-0.335662	1.564359
Br	-0.767698	-0.335662	-1.564359
F	0.469481	-2.070011	0.000000

$F_3N\cdots Cl^-$
E = -654.46
N_{imag} = 0

N	0.144541	0.589364	0.000000
F	0.167897	1.993453	0.000000
F	-0.668004	0.358828	1.048791
F	-0.668004	0.358828	-1.048791
Cl	0.749892	-2.592666	0.000000

$Cl_3N\cdots Cl^-$
E = -511.08
N_{imag} = 0

N	0.000000	0.000000	0.154715
Cl	0.000000	-1.411055	-0.891502
Cl	0.000000	1.411055	-0.891502
Cl	-2.230234	0.000000	0.823562
Cl	2.230234	0.000000	0.823562

$Br_3N\cdots Cl^-$
E = -485.71
N_{imag} = 0

N	0.125159	0.364584	0.000000
Br	-0.902876	-0.126707	1.559900
Br	-0.902876	-0.126707	-1.559900
Br	0.189215	2.380353	0.000000
Cl	1.480533	-2.100209	0.000000

$F_3N\cdots Br^-$

E **=** -645.10

N_{imag} = 0

N	0.028305	0.929630	0.000000
F	0.077148	2.324473	0.000000
F	-0.787979	0.707762	1.049194
F	-0.787979	0.707762	-1.049194
Br	0.705095	-2.487583	0.000000

$Cl_3N\cdots Br^-$

E **=** -501.99

N_{imag} = 0

N	0.137218	-0.290134	0.000000
Cl	-0.920571	-0.347397	1.426909
Cl	-0.920571	-0.347397	-1.426909
Cl	1.046788	-1.960939	0.000000
Br	0.436098	2.614233	0.000000

$Br_3N\cdots Br^-$

E **=** -475.97

N_{imag} = 0

N	0.000000	0.000000	0.042993
Br	0.000000	-1.583264	-1.132542
Br	0.000000	1.583264	-1.132542
Br	-2.080213	0.000000	1.074513
Br	2.080213	0.000000	1.074513

Table S5. Cartesian coordinates, bonding energies (in kcal mol^{-1}) and the number of imaginary vibrational frequencies (N_{imag}) for all stationary points of $D_2P^{\bullet}$, D_3P and $D_3P\cdots A^-$ structures, computed at ZORA-M06/QZ4P.

$F_2P^{\bullet}$
E = -437.52
N_{imag} = 0

P	0.000000	0.000000	0.449316
F	0.000000	1.193696	-0.580018
F	0.000000	-1.193696	-0.580018

$Cl_2P^{\bullet}$
E = -307.18
N_{imag} = 0

P	0.000000	0.000000	0.488705
Cl	0.000000	1.588518	-0.790998
Cl	0.000000	-1.588518	-0.790998

$Br_2P^{\bullet}$
E = -276.17
N_{imag} = 0

P	0.000000	0.000000	0.515621
Br	0.000000	1.721490	-0.858336
Br	0.000000	-1.721490	-0.858336

F_3P
E = -634.99
N_{imag} = 0

P	0.000000	0.000000	0.598083
F	-0.678515	1.175223	-0.178630
F	-0.678515	-1.175223	-0.178630
F	1.357030	0.000000	-0.178630

Cl_3P
E = -419.72
N_{imag} = 0

P	0.000000	0.000000	0.711971
Cl	-0.911235	1.578306	-0.236744
Cl	-0.911235	-1.578306	-0.236744
Cl	1.822470	0.000000	-0.236744

Br_3P
E = -370.81
N_{imag} = 0

P	0.000000	0.000000	0.773113
Br	-0.991177	1.716768	-0.242240
Br	-0.991177	-1.716768	-0.242240
Br	1.982353	0.000000	-0.242240

$F_3P\cdots F^-$
E = -820.73
N_{imag} = 0

P	0.471584	-0.045508	0.000000
F	-0.575038	-0.004777	-1.220655
F	-0.575038	-0.004777	1.220655
F	0.260247	-1.786014	0.000000
F	0.396623	1.706005	0.000000

$Cl_3P\cdots F^-$
E = -623.93
N_{imag} = 0

P	0.575347	-0.103428	0.000000
Cl	-0.764731	-0.261293	1.612478
Cl	-0.764731	-0.261293	-1.612478
Cl	0.171808	2.492628	0.000000
F	0.868878	-1.726052	0.000000

$Br_3P\cdots F^-$
E = -578.65
N_{imag} = 0

P	0.625146	-0.161380	0.000000
Br	-0.819389	-0.342368	1.744580
Br	-0.819389	-0.342368	-1.744580
Br	0.235596	2.685646	0.000000
F	0.976812	-1.759796	0.000000

$F_3P\cdots Cl^-$
E = -767.30
N_{imag} = 0

P	0.426576	-0.301072	0.000000
F	-0.607904	-0.213195	-1.190925
F	-0.607904	-0.213195	1.190925
F	0.370809	-1.947454	0.000000
Cl	0.357187	2.397614	0.000000

$Cl_3P\cdots Cl^-$
E = -561.45
N_{imag} = 0

P	0.497391	0.133951	0.000000
Cl	-0.816339	-0.139683	1.601529
Cl	-0.816339	-0.139683	-1.601529
Cl	0.123289	2.474218	0.000000
Cl	1.089662	-2.161122	0.000000

$Br_3P\cdots Cl^-$
E = -515.57
N_{imag} = 0

P	0.568754	0.030817	0.000000
Br	-0.856913	-0.245260	1.737601
Br	-0.856913	-0.245260	-1.737601
Br	0.212939	2.615147	0.000000
Cl	1.197970	-2.193831	0.000000

$F_3P\cdots Br^-$

***E* =** -755.35

N_{imag} = 0

P	0.413389	-0.521103	0.000000
F	-0.622566	-0.471654	-1.186509
F	-0.622566	-0.471654	1.186509
F	0.462055	-2.151576	0.000000
Br	0.238774	2.456137	0.000000

$Cl_3P\cdots Br^-$

***E* =** -547.76

N_{imag} = 0

P	0.409637	0.319838	0.000000
Cl	-0.898438	0.032750	1.597765
Cl	-0.898438	0.032750	-1.597765
Cl	0.087809	2.614891	0.000000
Br	1.089627	-2.206932	0.000000

$Br_3P\cdots Br^-$

***E* =** -501.72

N_{imag} = 0

P	0.492980	0.160480	0.000000
Br	-0.926680	-0.132607	1.735107
Br	-0.926680	-0.132607	-1.735107
Br	0.183894	2.691108	0.000000
Br	1.213018	-2.285483	0.000000

Table S6. Cartesian coordinates, bonding energies (in kcal mol^{-1}) and the number of imaginary vibrational frequencies (N_{imag}) for all stationary points of $D_2As^{\cdot}$, D_3As and $D_3As\cdots A^-$ structures, computed at ZORA-M06/QZ4P.

$F_2As^{\cdot}$
E = -406.24
***N*imag** = 0

As	0.000000	0.000000	0.523383
F	0.000000	1.291015	-0.632746
F	0.000000	-1.291015	-0.632746

$Cl_2As^{\cdot}$
E = -292.53
***N*imag** = 0

As	0.000000	0.000000	0.549067
Cl	0.000000	1.667356	-0.840659
Cl	0.000000	-1.667356	-0.840659

$Br_2As^{\cdot}$
E = -264.06
***N*imag** = 0

As	0.000000	0.000000	0.580135
Br	0.000000	1.795809	-0.896786
Br	0.000000	-1.795809	-0.896786

F_3As
E = -586.52
***N*imag** = 0

As	0.000000	0.000000	0.678088
F	-0.735339	1.273644	-0.207718
F	-0.735339	-1.273644	-0.207718
F	1.470677	0.000000	-0.207718

Cl_3As
E = -402.48
***N*imag** = 0

As	0.000000	0.000000	0.761728
Cl	-0.957050	1.657659	-0.278656
Cl	-0.957050	-1.657659	-0.278656
Cl	1.914100	0.000000	-0.278656

Br_3As
E = -357.61
***N*imag** = 0

As	0.000000	0.000000	0.826786
Br	-1.034028	1.790990	-0.273400
Br	-1.034028	-1.790990	-0.273400
Br	2.068057	0.000000	-0.273400

$F_3As\cdots F^-$
E = -784.31
***N*imag** = 0

As	0.519932	0.140398	0.000000
F	0.137352	2.001938	0.000000
F	-0.632615	-0.006025	1.317394
F	-0.632615	-0.006025	-1.317394
F	0.616005	-1.757348	0.000000

$Cl_3As\cdots F^-$
E = -609.25
***N*imag** = 0

As	0.594428	0.012026	0.000000
Cl	0.276405	2.577110	0.000000
Cl	-0.837011	-0.229511	1.691083
Cl	-0.837011	-0.229511	-1.691083
F	0.958406	-1.802055	0.000000

$Br_3As\cdots F^-$
E = -565.91
***N*imag** = 0

As	0.703181	-0.051608	0.000000
Br	0.295178	2.707101	0.000000
Br	-0.808690	-0.402281	1.819102
Br	-0.808690	-0.402281	-1.819102
F	1.224672	-1.819117	0.000000

$F_3As\cdots Cl^-$
E = -731.50
***N*imag** = 0

As	0.459413	0.275542	0.000000
F	0.217951	2.095210	0.000000
F	-0.700949	0.135775	1.294282
F	-0.700949	0.135775	-1.294282
Cl	0.695304	-2.308704	0.000000

$Cl_3As\cdots Cl^-$
E = -553.88
***N*imag** = 0

As	0.529968	0.132065	0.000000
Cl	0.280301	2.584045	0.000000
Cl	-0.898392	-0.144841	1.680286
Cl	-0.898392	-0.144841	-1.680286
Cl	1.215379	-2.235517	0.000000

$Br_3As\cdots Cl^-$
E = -510.10
***N*imag** = 0

As	0.649394	0.048438	0.000000
Br	0.289716	2.686734	0.000000
Br	-0.855424	-0.352654	1.811488
Br	-0.855424	-0.352654	-1.811488
Cl	1.563182	-2.211500	0.000000

$F_3As\cdots Br^-$

***E* =** -718.19

***N*$_{imag}$** = 0

As	0.424334	0.440561	0.000000
F	0.310043	2.257349	0.000000
F	-0.741888	0.360472	1.290175
F	-0.741888	0.360472	-1.290175
Br	0.583429	-2.375107	0.000000

$Cl_3As\cdots Br^-$

***E* =** -540.10

***N*$_{imag}$** = 0

As	0.452922	0.264327	0.000000
Cl	0.248881	2.692306	0.000000
Cl	-0.975203	-0.008603	1.677979
Cl	-0.975203	-0.008603	-1.677979
Br	1.227408	-2.288391	0.000000

$Br_3As\cdots Br^-$

***E* =** -497.12

***N*$_{imag}$** = 0

As	0.581753	0.122461	0.000000
Br	0.311113	2.742456	0.000000
Br	-0.929011	-0.251336	1.810385
Br	-0.929011	-0.251336	-1.810385
Br	1.564658	-2.321615	0.000000

Table S7. Cartesian coordinates, bonding energies (in kcal mol^{-1}) and the number of imaginary vibrational frequencies (N_{imag}) for all stationary points of $D_2Sb^{\cdot}$, D_3Sb and $D_3Sb\cdots A^-$ structures, computed at ZORA-M06/QZ4P.

$F_2Sb^{\cdot}$
E = -390.81
N N_{imag} = 0

Sb	0.000000	0.000000	0.567748
F	0.000000	1.403194	-0.729636
F	0.000000	-1.403194	-0.729636

$Cl_2Sb^{\cdot}$
E = -280.45
N_{imag} = 0

Sb	0.000000	0.000000	0.611682
Cl	0.000000	1.772166	-0.932213
Cl	0.000000	-1.772166	-0.932213

$Br_2Sb^{\cdot}$
E = -252.20
N_{imag} = 0

Sb	0.000000	0.000000	0.666436
Br	0.000000	1.899409	-0.965193
Br	0.000000	-1.899409	-0.965193

F_3Sb
E = -572.58
N_{imag} = 0

Sb	0.000000	0.000000	0.722162
F	-0.801030	1.387425	-0.286157
F	-0.801030	-1.387425	-0.286157
F	1.602060	0.000000	-0.286157

Cl_3Sb
E = -396.78
N_{imag} = 0

Sb	0.000000	0.000000	0.813112
Cl	-1.015350	1.758637	-0.366356
Cl	-1.015350	-1.758637	-0.366356
Cl	2.030699	0.000000	-0.366356

Br_3Sb
E = -352.22
N_{imag} = 0

Sb	0.000000	0.000000	0.902866
Br	-1.091026	1.889712	-0.337508
Br	-1.091026	-1.889712	-0.337508
Br	2.182051	0.000000	-0.337508

$F_3Sb\cdots F^-$
E = -781.38
N_{imag} = 0

Sb	0.595336	0.184347	0.000000
F	0.052902	2.147485	0.000000
F	-0.669109	-0.004238	1.445482
F	-0.669109	-0.004238	-1.445482
F	0.649524	-1.851555	0.000000

$Cl_3Sb\cdots F^-$
E = -610.69
N_{imag} = 0

Sb	0.656380	0.111787	0.000000
Cl	0.171576	2.715889	0.000000
Cl	-0.900766	-0.152291	1.805164
Cl	-0.900766	-0.152291	-1.805164
F	0.895401	-1.890987	0.000000

$Br_3Sb\cdots F^-$
E = -566.72
N_{imag} = 0

Sb	0.734407	0.059751	0.000000
Br	0.237165	2.857815	0.000000
Br	-0.915336	-0.254475	1.931449
Br	-0.915336	-0.254475	-1.931449
F	1.044699	-1.930373	0.000000

$F_3Sb\cdots Cl^-$
E = -727.75
N_{imag} = 0

Sb	0.565157	0.283126	0.000000
F	0.124801	2.238750	0.000000
F	-0.718724	0.095962	1.419111
F	-0.718724	0.095962	-1.419111
Cl	0.768715	-2.351540	0.000000

$Cl_3Sb\cdots Cl^-$
E = -555.78
N_{imag} = 0

Sb	0.614244	0.148534	0.000000
Cl	0.156546	2.699643	0.000000
Cl	-0.946801	-0.154570	1.788222
Cl	-0.946801	-0.154570	-1.788222
Cl	1.144570	-2.388602	0.000000

$Br_3Sb\cdots Cl^-$
E = -512.00
N_{imag} = 0

Sb	0.712180	0.101959	0.000000
Br	0.217989	2.839521	0.000000
Br	-0.937925	-0.268048	1.916640
Br	-0.937925	-0.268048	-1.916640
Cl	1.364108	-2.394354	0.000000

$F_3Sb\cdots Br^-$

***E* =** -713.71

N_{imag} = 0

Sb	0.533256	0.426676	0.000000
F	0.139622	2.383114	0.000000
F	-0.755540	0.253135	1.414293
F	-0.755540	0.253135	-1.414293
Br	0.765369	-2.403448	0.000000

$Cl_3Sb\cdots Br^-$

***E* =** -541.59

N_{imag} = 0

Sb	0.593711	0.220878	0.000000
Cl	0.123130	2.754101	0.000000
Cl	-0.963961	-0.105965	1.784919
Cl	-0.963961	-0.105965	-1.784919
Br	1.245475	-2.481383	0.000000

$Br_3Sb\cdots Br^-$

***E* =** -497.77

N_{imag} = 0

Sb	0.708896	0.154014	0.000000
Br	0.181578	2.869408	0.000000
Br	-0.933794	-0.252768	1.913972
Br	-0.933794	-0.252768	-1.913972
Br	1.508974	-2.494043	0.000000
